# Understanding the limitations of substrate degradation in bioelectrochemical systems

**DOI:** 10.3389/fmicb.2024.1511142

**Published:** 2025-01-06

**Authors:** Hannah Bird, Sharon Velasquez-Orta, Elizabeth Heidrich

**Affiliations:** School of Engineering, Newcastle University, Newcastle upon Tyne, United Kingdom

**Keywords:** microbial fuel cells, wastewater treatment, microbial metabolism, substrate degradation, mass transfer, kinetics

## Abstract

Microbial Fuel Cells (MFCs) are innovative environmental engineering systems that harness the metabolic activities of microbial communities to convert chemical energy in waste into electrical energy. However, MFC performance optimization remains challenging due to limited understanding of microbial metabolic mechanisms, particularly with complex substrates under realistic environmental conditions. This study investigated the effects of substrate complexity (acetate vs. starch) and varying mass transfer (stirred vs. non-stirred) on acclimatization rates, substrate degradation, and microbial community dynamics in air-cathode MFCs. Stirring was critical for acclimating to complex substrates, facilitating electrogenic biofilm formation in starch-fed MFCs, while non-stirred MFCs showed limited performance under these conditions. Non-stirred MFCs, however, outperformed stirred systems in current generation and coulombic efficiency (CE), especially with simple substrates (acetate), achieving 66% CE compared to 38% under stirred conditions, likely due to oxygen intrusion in the stirred systems. Starch-fed MFCs exhibited consistently low CE (19%) across all tested conditions due to electron diversion into volatile fatty acids (VFA). Microbial diversity was higher in acetate-fed MFCs but unaffected by stirring, while starch-fed MFCs developed smaller, more specialized communities. Kinetic analysis identified hydrolysis of complex substrates as the rate-limiting step, with rates an order of magnitude slower than acetate consumption. Combined hydrolysis-fermentation rates were unaffected by stirring, but stirring significantly impacted acetate consumption rates, likely due to oxygen-induced competition between facultative aerobes and electrogenic bacteria. These findings highlight the trade-offs between enhanced substrate availability and oxygen-driven competition in MFCs. For real-world applications, initiating reactors with dynamic stirring to accelerate acclimatization, followed by non-stirred operation, may optimize performance. Integrating MFCs with anaerobic digestion could overcome hydrolysis limitations, enhancing the degradation of complex substrates while improving energy recovery. This study introduces novel strategies to address key challenges in scaling up MFCs for wastewater treatment, bridging the gap between fundamental research and practical applications to advance environmental systems.

## 1 Introduction

Microbial fuel cells (MFCs) are an emerging waste treatment technology. Their ability to convert chemical energy in wastewater directly into electrical energy in ambient operating conditions means they are being seen as an alternative to traditional energy-intensive wastewater treatment methods such as activated sludge (AS) (Trapero et al., 2017). Over the last few decades, considerable progress has been made by the research community to help improve the performance of MFC systems. Developments include research into various reactor designs and configurations (Janicek et al., 2014), electrode materials and surface modifications (Sonawane et al., 2017; Agrahari et al., 2022), electron transfer mechanisms (Patil et al., 2012; Prathiba et al., 2022), substrate types (Pant et al., 2010; Pandey et al., 2016; Sonawane et al., 2022), operational conditions (Zafar et al., 2022), pilot-scale studies (Bird et al., 2022), as well as microbial communities and biofilm performance (Cao et al., 2019; Angelaalincy et al., 2018). Despite efforts into enhancing the performance of MFCs, a lack of understanding on what the fundamental microbial mechanisms are under realistic environmental conditions and with complex waste streams, hinders this technology’s optimization. An important facet of MFCs is that through the generation of current, they offer a unique window into examining in real time the processes that occur within the bioelectric system and possibly anaerobic metabolism more widely.

**Figure 1:**
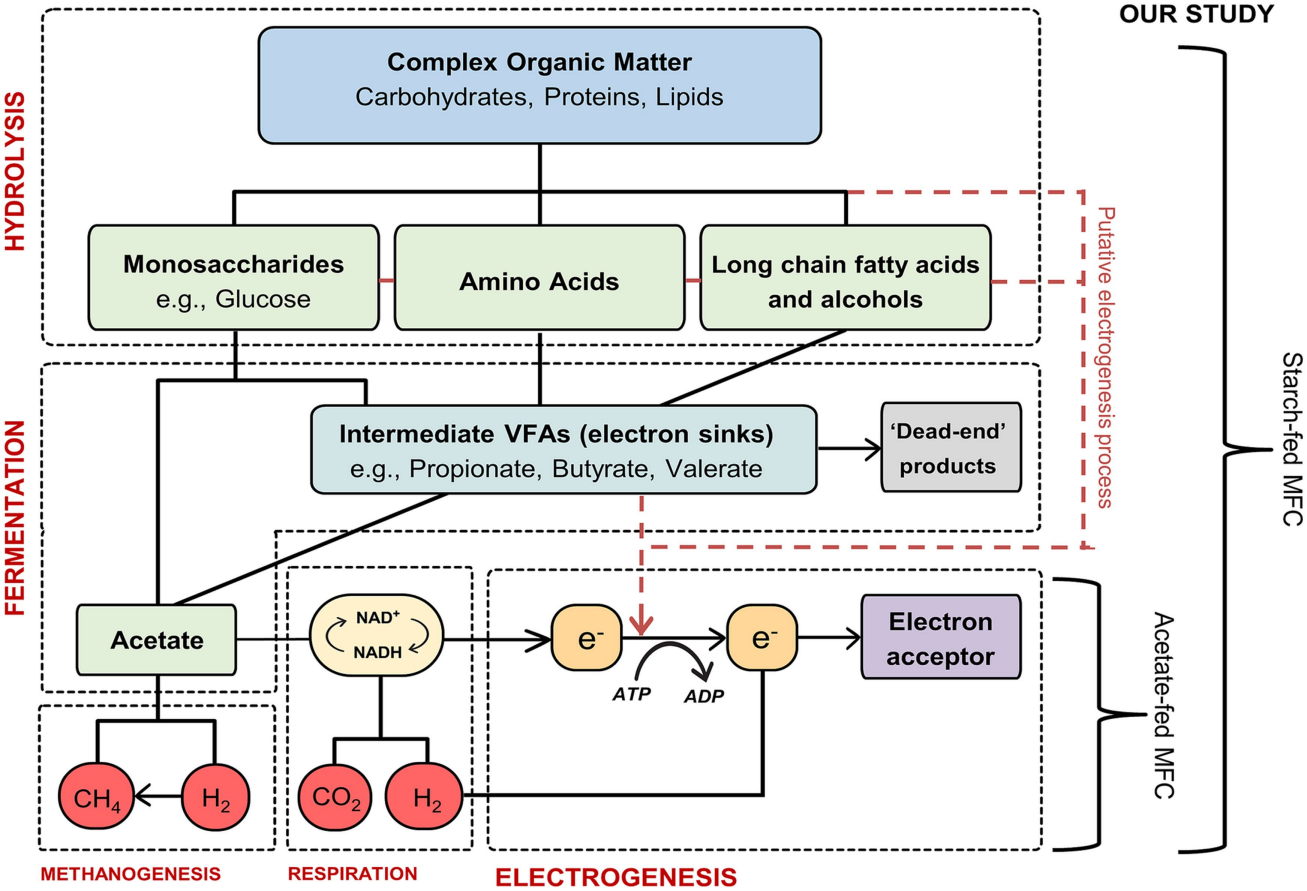
Schematic diagram showing the overall stages in the substrate degradation pathway of complex organic matter in MFCs. Adapted from Velasquez-Orta et al. (2011) and Ebadinezhad et al. (2019).

It is generally assumed the degradation pathways and rates of complex organic compounds in MFCs are similar to those in anaerobic digestion (AD) (Metcalf and Eddy, 2003). Complex compounds, e.g., starch, a polymeric carbohydrate, are used as fuel by bacterial cells and undergo hydrolysis into simpler molecules such as glucose. Following hydrolysis, these simpler molecules are further degraded via fermentation under anaerobic conditions, producing carbon dioxide, hydrogen, volatile fatty acids (VFAs, e.g., acetate, butyrate, and propionate) and other organic products (Torres et al., 2007). The final step in this process is electrogenesis, during which electroactive bacteria (EAB) consume acetate, releasing electrons and protons. The electrons produced during electrogenesis are transferred to the anode and can be harnessed as energy (Logan, 2009). Figure 1 illustrates the degradation pathway of complex organic molecules and the electron flow in MFCs. Previous research has shown that coulombic efficiencies (CE) when using simple substrates such as acetate are significantly higher when compared to more complex substrates (Velasquez-Orta et al., 2011; Torres et al., 2007; Thygesen et al., 2009; Lee et al., 2008). A study by Chae et al. (2009) compared the performance of different substrates that varied in complexity and reported a higher CE when using the simple substrate acetate (72.3%) but a lower microbial community diversity when compared to the more complex substrate glucose (15.0% CE). Unlike in AD, where the final step of the degradation process is completed by methanogens, in MFCs the anode respiring bacteria act as the electron acceptors and consume the VFAs, transmitting electrons to the anode (electrogenesis). However, competing microorganisms such as secondary fermenters and methanogens can also utilize these VFAs, resulting in electron sinks (Jung and Regan, 2011). In addition to these sinks, (which ultimately reduce the amount of electrons available and thus the overall CE), there are also stages within this degradation pathway that are known bottlenecks, i.e., rates of certain pathways are slower than others. This will reduce the current produced, (measured as electrons per second), in an MFC.

In classical AD systems, the conversion of organic wastes via hydrolysis is considered as the rate-limiting step, with reported first-order rate constants for hydrolysis ranging between 0.025 and 0.047 d^−1^ (Christ et al., 2000; Vavilin et al., 2001). Consequently, significant effort has been dedicated in recent years to enhance the performance of AD using various pre-treatment techniques (Mata-Alvarez et al., 2000). A study reported an increase in the hydrolysis rate constant by a factor of 1.5–10 when a pre-hydrolysis treatment was applied (Zhang et al., 2016). Hydrolysis has also been reported as the rate-limiting step in MFCs. A study by Velasquez-Orta et al. (2011) using single chambered MFCs fed with either acetate, glucose or starch reported a combined hydrolysis-fermentation rate that was an order of magnitude lower than the rates of both fermentation and acetate consumption. This was the first time the substrate degradation rate constants have been estimated in MFCs. However, this study was conducted under fully stirred conditions, eliminating mass transfer limitations. The integration of MFCs with anaerobic systems such as AD, has been explored as a solution to enhance the treatment rates of complex wastes (Bajracharya, 2020). Studies have demonstrated the feasibility of a synergistic relationship between AD and MFCs (Ardakani and Badalians Gholikandi, 2020; Gude, 2016). For instance, significant increases in power outputs were reported in an MFC system that incorporated a pre-fermentation step via an anaerobic reactor (Kannaiah Goud and Venkata Mohan, 2011). However, if a pre-treatment step were to be implemented at full-scale operation, these performance improvements would need to be balanced against the additional costs of tanks and land area.

In addition to treatment rates, mass transfer has been identified as a factor that can significantly influence the performance of MFCs (Yang et al., 2021). While increasing the hydrodynamic flow of a system via mixing is considered an effective technique to increase mass transfer and in turn MFC performance, it requires an additional energy input which can be costly and may in fact offset the improved power generation, resulting in negative energy balances (Zou and He, 2018; Pan et al., 2019). Many studies fail to report energy consumption of the pumping, making it challenging to evaluate if these systems are truly ‘energy efficient’. However, a study by Zhang et al. (2013) measured the energy balance of a bench-scale air-cathode tubular MFC that used recirculation pumps to promote anolyte mixing. The system produced a total of 0.026 kWh/m^3^ of energy but consumed 0.024 kWh/m^3^, resulting in a net energy production of only 0.002 kWh/m^3^. To put this into context, a typical household in the UK will use 7.5 kWh/day (Milligan, 2023) or 30 kWh/day in the United States (Energy Information Administration, 2022).

Hydrodynamics also play a vital role in shaping biofilms. Recent studies have examined electroactive biofilms and demonstrated that shear-stress, i.e., increased substrate disturbance, influences the composition anodic biofilms (Godain et al., 2023, 2024; Jones and Buie, 2019). A study by Godain et al. (2023) revealed that higher shear stresses favored the adhesion of EAB such as *Geobacter* onto anode surfaces, however, overtime this initial positive effect of EAB selection decreased. They suggested future research should focus on the impact of long-term shear stress exposure on anodic biofilm development and physical structure. Substrate types are also known to impact microbial communities in MFCs, in terms of composition, which in turn influences MFC efficiency (Zhang et al., 2011; Chae et al., 2009; Catal et al., 2008). Studies show that MFCs fed with substrates such as glucose and complex starch, have increased bacterial diversity when compared to simple substrates such as acetate (Chae et al., 2009; Pant et al., 2010). These more diverse communities can comprise of non-electrogenic and syntrophic microbial species which can also contribute to the degradation process by metabolizing the soluble fermentation end products, in turn indirectly facilitating MFC power production.

This study aims to elucidate the competing metabolic pathways and bottlenecks within complex biological systems such as MFCs to inform the design of reactors and operational conditions that maximize their efficacy for treating complex wastes. To achieve this, the effects of substrate complexity (acetate vs. starch) and varying mass transfer conditions (stirring vs. non-stirring) on acclimatization rates, substrate degradation rates, and anodic microbial community selection in MFCs were investigated. It was hypothesized that MFCs fed with complex substrates (starch) would perform worse in terms of current production but would present greater microbial community diversity due to the increased number of degradation processes carried out by different bacteria. Additionally, it was hypothesized that MFC performance would improve under stirred conditions due to reduced mass transfer limitations, though microbial diversity might decrease as shear-stress would result in thinner, sturdier biofilms. By systematically varying these conditions, this research provides valuable insights into how reactor design and operational strategies influence the performance of bioelectrochemical systems. The findings aim to bridge the gap between fundamental research and real-world applications, offering solutions to enhance the degradation of complex substrates and improve wastewater treatment efficiency. This work introduces novel strategies to address key challenges in scaling up MFCs for practical applications, offering approaches to improve energy recovery and waste treatment efficiency in environmental systems.

## 2 Materials and methods

### 2.1 Reactor configuration

**Figure 2:**
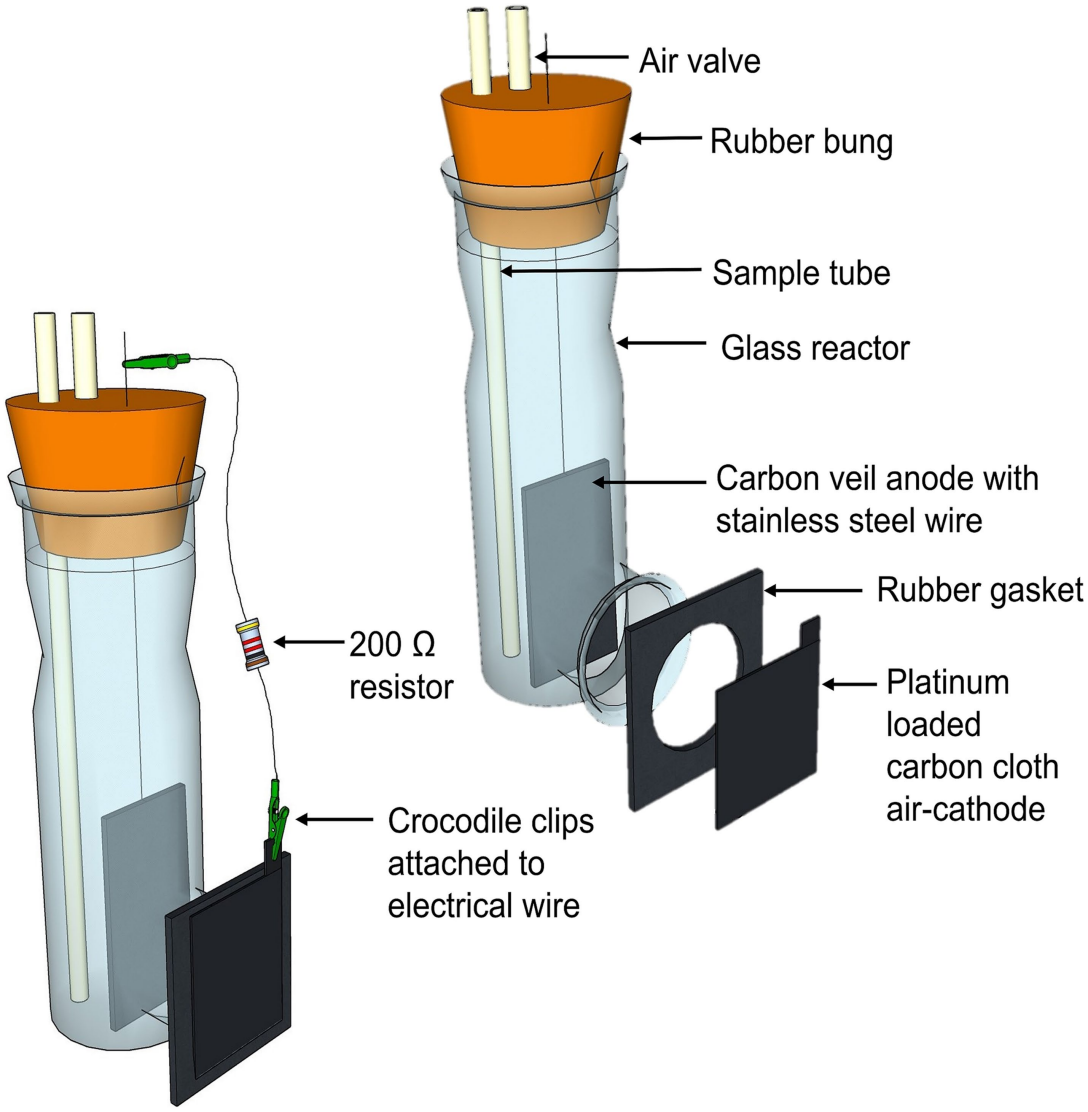
3D sketch of the single-chambered air-cathode MFC design (left) and in an exploded view to show the individual components (right).

Six single-chambered glass MFCs were used, each with a total anodic volume of 330 mL (Figure 2). A carbon veil anode (OPTIVEIL, Technical Fibre Products, UK) with a projected surface area of 27.2 cm^2^ was used. The air-cathode electrode had a surface area of 12.6 cm^2^ and was a 0.5 mg/cm^2^ 20% Platinum loaded Vulcan carbon cloth (Fuel Cell Store^®^, Texas, United States). The cathode cloth was glued onto a rubber gasket and then glued to the glass reactor using an epoxy resin (Gorilla Glue Ltd., UK). The electrodes were positioned 4 cm apart and were connected to a 200 Ω resistor using 0.6 mm^2^ stainless steel wire (Clarke © Tools, Chronos Ltd., Dunstable, UK). All wire connections were soldered to maintain high connection. The anodic chamber was sealed using a rubber bung, containing both an air valve and sample port made from Tygon Tubing (Saint-Gobain Tygon^®^ S3™ E-3606), to ensure the anodic biofilm remained undisturbed when the anolyte was being removed.

### 2.2 Reactor inoculation and operation

MFCs were inoculated with a 330 mL 50:50 mix of fresh return sludge liquor (RSL) and one synthetic substrate (either acetate or starch at a concentration of 500 mgCOD/L) that consisted of: 0.528 g/L NaH_2_PO_4_∙H_2_O, 0.984 g/L Na_2_HPO_4_, 1 g/L NaC_2_H_3_O_2_ or 0.5 g/L of starch, 1.5 mL of both vitamin and trace element solution (ATCC., Teddington, UK) (chemicals were analytical grade, Sigma-Aldrich). The RSL was collected from Howdon Sewage Treatment Works, Newcastle, UK (Northumbrian Water Ltd). RSL, a high-strength waste stream with COD values of 1,500–6,000 mg/L (Leicester, 2020) contains a high proportion of soluble organic matter and is typically recycled to the beginning of the treatment works.

Four distinct MFC setups were designed to evaluate the combined effects of substrate complexity and mass transfer conditions:

1. Non-stirred MFC fed with acetate: Represents a simplified system, similar to small batch experiments commonly reported in MFC literature. It isolates the acetate consumption process under minimal external influences.
2. Stirred MFC fed with acetate: Introduced controlled mixing to reduce mass transfer limitations, providing insight into the acetate consumption metabolic pathway under optimized conditions.
3. Non-stirred MFC fed with starch: Simulates lab-scale batch experiments using a more complex substrate comparable to real wastewater, emphasizing challenges in substrate degradation (hydrolysis-fermentation) under diffusion-limited conditions.
4. Stirred MFC fed with starch: Represents the most realistic configuration, resembling a tank system treating a continuous flow of wastewater or operating under continuous mixing.

These configurations provided a systematic approach to evaluate how substrate complexity and mixing influence MFC performance.

The inoculums were sparged with ultra-high purity (UHP) nitrogen gas (99.998%) for 20 min to remove any traces of oxygen. To ensure sufficient MFC anode enrichment and adaptation of electroactive biofilms for biofilm formation, the system was operated in batch mode until a reproducible current was produced over three consecutive cycles. For each substrate, three MFCs were tested under each stirring condition. For the stirred MFCs, a mixing speed of 100 rpm was used, chosen to optimize substrate mass transfer without causing excessive shear stress that could damage biofilms. This speed also avoids biomass washout or disruption of microbial consortia, common at higher agitation levels (Zhong et al., 2021; Johnson et al., 2021). After enrichment, the MFCs were fed with 330 mL of the synthetic substrate used in the inoculation phase (150 mg COD/L for acetate and 250 mg COD/L for starch). These concentrations were determined based on preliminary experiments designed to optimize batch cycle durations within 1 week, ensuring full depletion of the organic substrate while allowing for consistent and evenly spaced sampling intervals. During the feeding process, anaerobic conditions were maintained by connecting a gas bag filled with N_2_ gas to the reactor to create an oxygen-free headspace. The valve and sample port were sealed with a tube clamp to prevent oxygen intrusion. Acetate-fed MFCs were placed in an incubator and operated at 22°C for 1 month under both stirred and non-stirred conditions. Starch-fed MFCs were operated sequentially: stirred MFCs were run first, followed by non-stirred MFCs using the same biofilms. This approach ensured that degradation rates for non-stirred starch-fed systems could still be calculated, as they produced minimal currents after the enrichment cycles. Voltage and current were continuously monitored at one-minute intervals using a data acquisition system (PICO Data Logger, UK). During batch cycles, no more than 10% of the total MFC volume (33 mL) was removed when analyzing the anolyte.

### 2.3 Chemical measurements

The following tests were conducted to chemically characterize the anolyte: chemical oxygen demand (COD), pH, conductivity and dissolved oxygen (DO). These were measured at both the beginning and end of each test. COD was measured using the HACH-Lange LCK 114 photometric test kits, according to standard methods. The Jenway 3310 digital pH probe and the HACH HQ30d conductivity and DO probes were used to measure these parameters. Samples for volatile fatty acid (VFA) analysis were filtered through a 0.2 μm syringe filter and measured by ion chromatography (Thermo Scientific Dionex Aquion IC system, ICE-AS1 column; mobile phase, 1 mM heptafluorobutyric acid; flow rate, 0.16 mL/min; backpressure ∼700 psi and a 25-μL capacity sample loop). The detection limit was 0.2 mg/L for lactic, formic, acetic, propionic and butyric acids. Samples were withdrawn from the MFCs daily, every few hours, with more sample points being taken at the beginning of the experiment (within the first 24 h) and were stored at −20°C. To ensure the interference of carbonate was minimized, 5 μL of concentrated hydrochloric acid was added to the samples.

### 2.4 Microbial community analysis

The microbial community for each substrate was analyzed. At the end of the experiments a 15 mm^2^ section from the central section of each anode was removed under aseptic conditions. The biofilm samples were preserved in 20% glycerol and stored in a freezer at −20°C. The microbial communities were analyzed by extracting DNA using the DNeasy PowerLyzer PowerSoil kit (Qiagen, UK) as per the manufacturer’s instructions and underwent sequencing at NU-OMICS (Northumbria University, UK). The Earth Microbiome 16S amplicon protocol (v2) was used to target the hypervariable V4 region of the 16S rRNA gene (Caporaso et al., 2011) and amplify using primers 515F (GTGYCAGCMGCCGCGGTAA) (Parada et al., 2016) to 806R (GGACTACNVGGGTWTCTAAT) (Apprill et al., 2015). Sequencing was performed on the Illumina MiSeq platform with V2 500-bp cycle chemistry (Illumina, UK). The following optimized thermocycler conditions were used: initial denaturation at 94°C for 3 min; 35 cycles (denaturation at 94°C for 45 s; annealing at 50°C for 60 s; extension at 72°C for 90 s); and a final extension at 72°C for 10 min. The DADA2 pipeline was used for amplicon sequence variant (ASV) selection (Callahan et al., 2016) and the SILVA rRNA gene database (v 132) was used to assign ASVs to family level (Yilmaz et al., 2014). Downstream analysis was carried out using the R package phyloseq (McMurdie and Holmes, 2013). To normalize the data, samples were rarefied to an equal sampling depth using the R function rarefy_ even_sampling_depth.

### 2.5 Calculations

Current density, COD removal efficiency, coulombic efficiency (CE), electron equivalents (e^−^eq) and degradation rates were calculated as per the equations below.

Voltage outputs were monitored using a PICO logger data acquisition system and were converted into current density based on the projected surface area of the anode. The current can be calculated as follows according to Ohms law:


$$I=\frac{V}{R}$$

where: *I* is the current (A); *V* is the voltage (volts); and *R* is the resistance (*Ω*).

The current density was then calculated as follows:


$$J=\frac{I}{A}$$

where: *J* is the current density (A/m^2^); and *A* is the projected anode surface area (m^2^).

The chemical oxygen demand (COD) is a measure of the amount of oxygen required to chemically oxidize the organic material in each sample (mg/L of oxygen), (Tchobanoglous et al., 2003). The COD removal efficiency (%) was calculated as follows:


$$\text{COD}\;\text{removal}=\frac{\Delta \text{COD}}{S_{o}}\times 100$$

where: ΔCOD is the difference in COD concentrations between the influent and effluent (mgCOD/L); and *S_o_* is the influent substrate concentration (mgCOD/L).

Coulombic efficiency (CE) represents the conversion of organic substrates into electrical charge as per the equation below, given by Logan (2008):


$$CE=\frac{M_{s}I}{\text{Fbq}\Delta \text{COD}}$$

where: *CE* is the coulombic efficiency (%); *M_s_* is the molecular weight of oxygen (32 g/mol); *I* is the current (A); *F* is the Faraday’s constant (96,485 C/mol e^–^); *b* is the number of electrons exchanged per mole of oxygen (4 mol e^−^ mol O_2_); *q* is the volumetric flow rate (L/s); and *ΔCOD* is the difference in the influent and effluent COD (g/L).

The electron equivalent (e^−^eq) of a molecule represents the amount of substance that releases 1 mole e^−^ following complete oxidation (Zhao et al., 2020), and was calculated using the equation below:


$$S_{e}=S_{m}\times N$$

where: *S_e_* is the e^−^eq of molecules (e^−^ mmol/L); *S_m_* is the concentration of molecules (mmol/L); and *N* is the number of electrons released per molecule when mineralized to CO_2_, protons, and electrons (Supplementary Table S1).

The rates of the different degradation pathways: hydrolysis-fermentation (conversion of starch to acetate); fermentation (glucose to acetate); and acetate consumption were calculated assuming a first-order reaction *(R → P)* (Velasquez-Orta et al., 2011). The rate equation for this type of reaction is as follows: *rate = −k [R]*, where *k* is the kinetic rate constant, *R* is the reactant concentration and *P* is the product concentration. *k* was obtained from a linear plot of time *(t)* vs. *R* (Supplementary Figure S1), as per the equation below:


lnR=−kt+lnRo

The consumption of starch was determined by subtracting the concentration of VFA produced through time from the starting substrate concentration (250 mg/L) and was assumed that the VFA production rate was proportional to the VFA accumulation rate.

## 3 Results

### 3.1 Influence of substrate complexity and mass transfer on MFC performance

**Figure 3:**
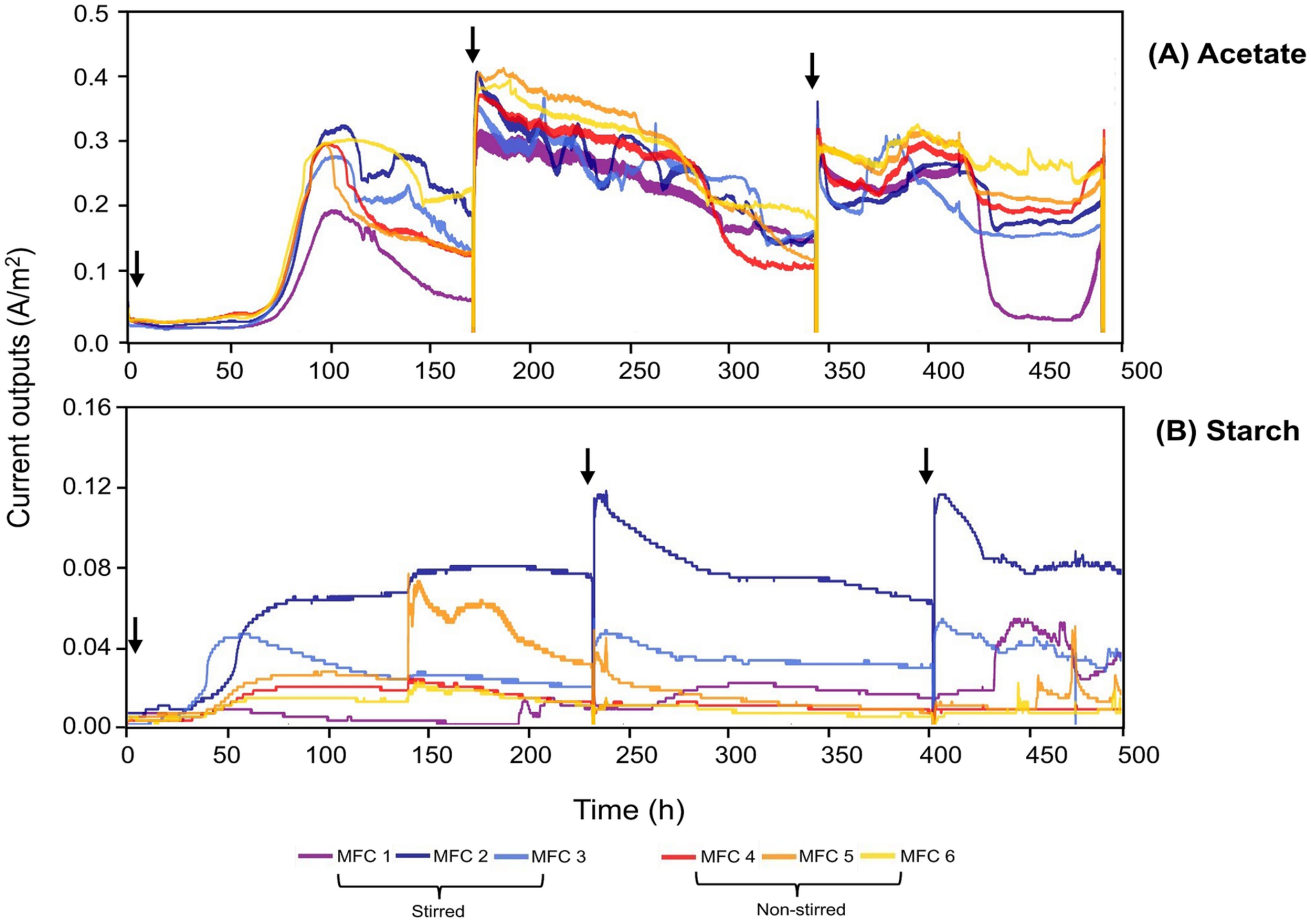
Enrichment phase for acetate-fed **(A)** and starch-fed MFCs **(B)**. A 50/50 mix of wastewater (RSL) and a synthetic substrate (500 mgCOD/L) was used for three enrichment cycles. Arrows indicate the addition of fresh inoculant. Note the different scale on the y-axis.

The enrichment phase of the MFCs can be seen in Figure 3. Both stirred and non-stirred acetate-fed MFCs acclimatized at a time of ~65 h and the rise in current was consistent among all replicas (Figure 3A). By the final acclimatization cycle, the MFCs were relatively stable in terms of current output. The starch-fed MFCs started producing current considerably earlier, ~35 h after inoculation (Figure 3B). However, their current outputs were less consistent compared to those fed acetate. In particular, the non-stirred starch-fed MFCs exhibited greater variability in terms of current output, and after the final round of enrichment, minimal currents were produced and the MFCs were determined as non-viable for the subsequent experiments. There was a significant difference between the peak currents produced by the two substrates during the enrichment phase (*p* < 0.001, *t-*test), with acetate-fed MFCs reaching a peak current of 0.40 ± 0.03 A/m^2^ at 180 h compared to 0.05 ± 0.04 A/m^2^ at 230 h for the starch-fed MFCs.

**Figure 4:**
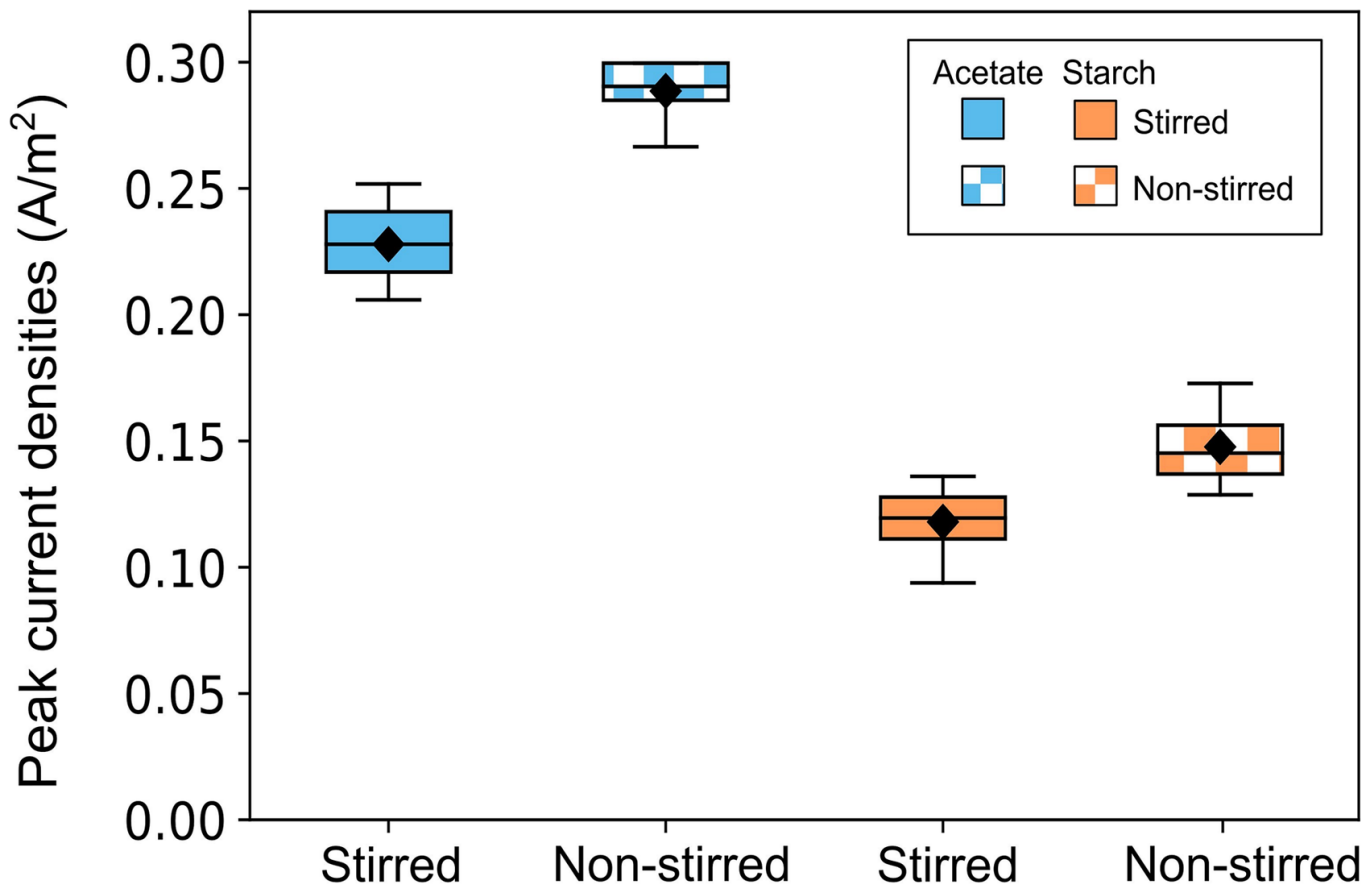
Box plot showing the maximum current outputs obtained by acetate-fed and starch-fed MFCs across all batch runs. Data represents three batch cycles for acetate (*n* = 9) and two batch cycles for starch (*n* = 6) under each stirring condition. Error bars denote standard deviation (SD).

Once MFC enrichment was complete (20 days), the influence of substrate complexity and mass transfer conditions on MFC performance was investigated. Acetate-fed MFCs achieved significantly higher average peak currents (0.26 ± 0.03 A/m^2^) compared to starch-fed MFCs (0.13 ± 0.02 A/m^2^) (*p* < 0.001, *t*-test) (Figure 4). Non-stirred MFCs consistently outperformed their stirred counterparts, achieving peak currents of 0.29 ± 0.01 A/m^2^ and 0.15 ± 0.02 A/m^2^ for acetate and starch-fed MFCs, respectively. In contrast, stirred MFCs recorded lower peak currents of 0.23 ± 0.02 A/m^2^ for acetate (*p* < 0.001, *t*-test) and 0.12 ± 0.02 A/m^2^ for starch (*p <* 0.05, *t*-test).

The acetate-fed MFCs achieved the highest COD removal efficiencies, ranging from 71 to 73%, significantly higher than the 47–69% observed in the starch-fed MFCs (*p* < 0.001, *t*-test) (Table 1). Among the acetate-fed systems, stirring had minimal impact on COD removal, with stirred MFCs removing only 2% more COD than their non-stirred counterparts, a difference that was not statistically significant (*p* > 0.05, *t*-test). In contrast, the starch-fed MFCs showed a significant difference in COD removal between mixing conditions where non-stirred MFCs removed 22% more COD than stirred MFCs (*p* < 0.001, *t*-test). Both COD removal and current production contribute to the CE of MFCs. In the acetate-fed systems, the non-stirred MFCs demonstrated a significantly higher CE, 28% greater than that of the stirred MFCs (*p* < 0.001, *t*-test). This was not the case in the starch-fed systems, where the lower current producing MFCs (under stirred conditions) also removed less COD, resulting in an identical CE to the non-stirred MFCs (19%) (Table 1).

**Table 1:** MFC performance with varying substrate complexities and stirring conditions.

Substrate and stirring condition	COD removal (%)	CE (%)
Acetate		
Stirred	73 ± 1.8	38 ± 9.2
Non-stirred	71 ± 3.3	66 ± 9.1
Starch		
Stirred	47 ± 4.8	19 ± 6.8
Non-stirred	69 ± 1.2	19 ± 3.9

Values represent the mean of replicates (acetate: *n* = 9, and starch: *n* = 6), with ± indicating the SD.

### 3.2 Influence of substrate complexity and mass transfer on VFA production and consumption in MFCs

**Figure 5:**
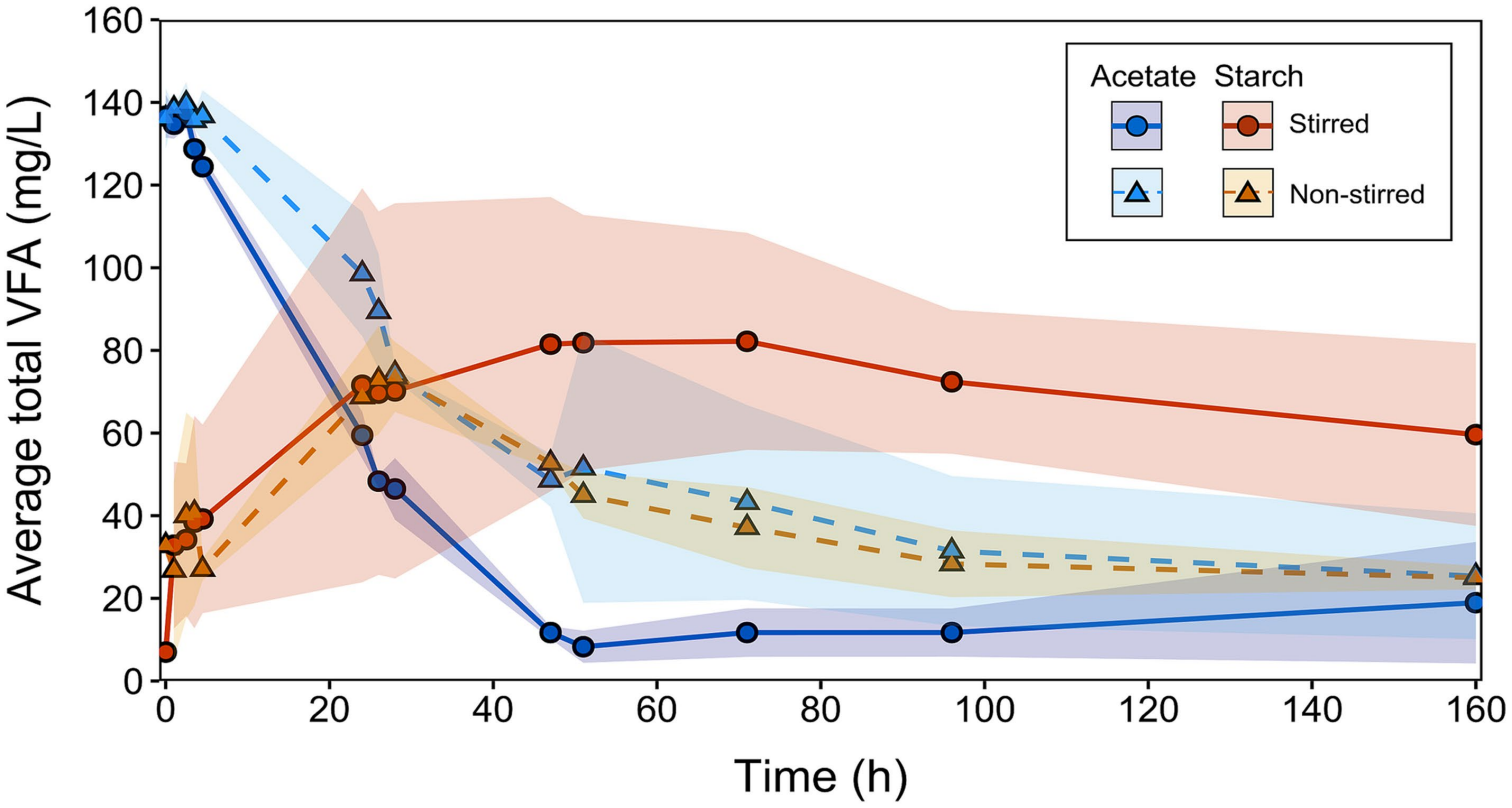
Combined total VFA production and consumption for acetate-fed and starch-fed MFCs across all batch runs. Solid lines with circular symbols represent stirred MFCs, while dashed lines with triangular symbols represent non-stirred MFCs. Data represents the mean of replicates (acetate: *n* = 9, and starch: *n* = 6). Error bands indicate the SD, reflecting variability in VFA concentrations between sequential runs (see Supplementary Figure S2).

**Figure 6:**
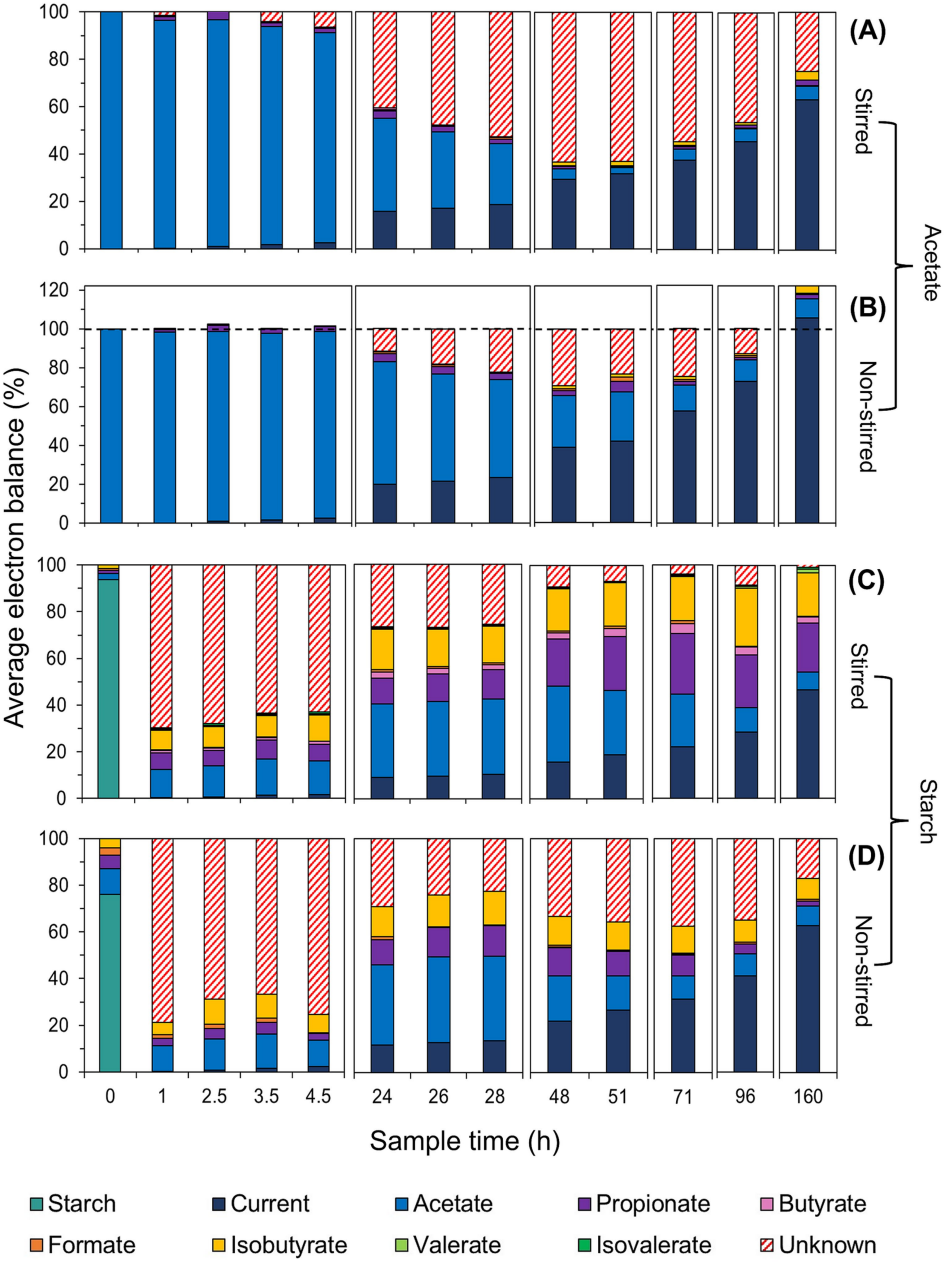
Electron distribution at the time of VFA sampling for acetate-fed **(A,B)** and starch-fed MFCs **(C,D)** under stirred **(A,C)** and non-stirred conditions **(B,D)**. The electron balance represents the percentage of electrons from the initial acetate or starch feed at different sampling times, based on calculated electron equivalents (see Supplementary Table S2 for a detailed breakdown of electron balance percentages and SD).

The performance of the MFCs was evaluated by monitoring the generation and consumption of VFAs under stirred and non-stirred conditions across all batch runs (Figure 5). By combining VFA concentrations along with current outputs, electron equivalent (e^−^eq) balances were calculated for each MFC condition. This enabled the mapping of electron flows within the systems, providing insight into energy losses (Figure 6).

A clear contrast between the different stirring conditions was seen in the MFCs using acetate. The non-stirred MFCs exhibited a slower rate of acetate consumption in the first 24 h (2.02 mg/L/h) compared to the stirred MFCs (3.46 mg/L/h) (Figure 5). When translated into e^−^eq (where 100% represents all available electrons in the system), 63 ± 13% and 39 ± 5% of electrons from the initial acetate feed were utilized for substrate consumption, in the non-stirred and stirred acetate-fed MFCs, respectively (Figures 6A,B). Despite the stirred MFCs having an overall faster rate of acetate consumption in the first 24 h, they had a much slower rate of charge transfer compared to non-stirred MFCs (16 ± 1% of the electrons from the initial acetate feed were used for current production compared to 20 ± 1% in the non-stirred MFCs). After 71 h of operation, VFA analysis shows marginal quantities of acetate accumulation in the stirred MFCs (Figures 5, 6A). This was not the case in the non-stirred MFCs, where acetate was constantly being consumed. Both systems accumulated small amounts of other VFAs such as propionate and isobutyrate.

Large fractions of “unknown” electrons or “sinks” (represented by red hatch lines in Figure 6) were observed in the acetate-fed MFCs, particularly under stirred conditions. These electron sinks increased after the initial phase of high current production and acetate consumption in both stirred and non-stirred MFCs, peaking after 48 h of operation. At this point, over half of the initial acetate feed in terms of electrons was unaccounted for in the stirred MFCs (63 ± 4%), compared to 30 ± 11% in non-stirred MFCs. However, electron balance closures show this fraction decreased to 25 ± 3 and 0%, respectively, with current being the primary electron sink in both systems. Notably, the electron closures in non-stirred MFCs exceeded 100%, reaching 122 ± 17%, with current contributing 105 ± 8% of the total (Figure 6B).

The VFA analysis of the starch-fed MFCs show that minimal amounts of acetate accumulated during the first 20 h despite both the stirred and non-stirred MFCs reaching a maximum current during this time. At 4.5 h, the acetate started to slowly accumulate at a rate of 0.96 mg/L/h for stirred MFCs and 0.92 mg/L/h for non-stirred. VFA concentrations can be seen to peak at 28 h in the non-stirred MFCs followed by a sudden decrease. The acetate accumulation during this time accounted for 32 ± 19% and 36 ± 6% of the initial starch feed in terms of electron balance for stirred and non-stirred MFCs, respectively. However, acetate concentrations slowly decreased again as other products formed, e.g., propionate, butyrate and isobutyrate (known to be “dead-end” products) (Figures 6C,D). After 48 h of operation, the VFA concentrations in the stirred starch-fed MFCs plateaued and then slowly decreased from 71 h onwards. Despite VFA analysis showing that stirred MFCs accumulated more of these ‘dead-end’ products, the ability of the MFCs to convert these into electrons for current production was low when compared to non-stirred MFCs, e.g., the closures of the electron balances for current production were 47 ± 9% for stirred and 63 ± 3% for non-stirred MFCs. These electron closures were lower than those of the acetate-fed MFCs which had released 63 and 105% electrons for current production in stirred and non-stirred MFCs, respectively.

A large fraction of unknown electrons was observed in the starch-fed MFCs, particularly under non-stirred conditions. This was likely due to the reactor volume preventing accurate measurements of carbohydrates (starch) and glucose. Similar to the acetate-fed MFCs, electron sinks in the starch-fed MFCs decreased over time, with electron closures revealing only 1 ± 5% and 17 ± 9% of unaccounted electrons in stirred and non-stirred MFCs, respectively. Another major electron sink in these air-cathode systems was oxygen intrusion, as measured using dissolved oxygen probes inside the reactor. Stirred MFCs drew in 0.14 mg/L of oxygen per hour from the air-cathode, while non-stirred MFCs drew in eight times less oxygen (0.017 mg/L/h) (Supplementary Figure S3B). To put this into perspective, there was enough oxygen in the stirred systems to consume 15 and 9% of the COD in the acetate and starch-fed MFCs, respectively, compared to only 1.8 and 1% in their non-stirred counterparts (see Supplementary material for calculation details).

The rates of the different degradation pathways were calculated based on the obtained VFA concentrations (Figure 5) and can be seen in Table 2 below. The hydrolysis-fermentation rates (conversion of starch to acetate) for stirred (*k* = 0.0034 ± 0.0027 h^−1^) and non-stirred MFCs (*k* = 0.0045 ± 0.0013 h^−1^) were an order of magnitude lower than the calculated rate constant for acetate consumption (includes electrogenesis as well as aerobic acetate metabolism) (*k* = 0.0411 ± 0.0038 h^−1^ and 0.023 ± 0.0095 h^−1^, for stirred and non-stirred MFCs, respectively). There were no significant differences in the hydrolysis-fermentation rates between stirred and non-stirred MFCs (*p* > 0.05, *t*-test). However, there was a significant difference in the rate of acetate consumption between the two stirring conditions (*p* < 0.05, *t*-test).

**Table 2:** The calculated substrate degradation rate constants for stirred and non-stirred MFCs.

Stirring condition	Hydrolysis-fermentation rate constant	Acetate consumption rate constant
Stirred	*k* = 0.0034 ± 0.0027 h^−1^	*k* = 0.0411 ± 0.0038 h^−1^
Non-stirred	*k* = 0.0045 ± 0.0013 h^−1^	*k* = 0.023 ± 0.0095 h^−1^

Values represent the mean of replicates (acetate: *n* = 9, and starch: *n* = 6), with ± indicating the SD.

### 3.3 Influence of substrate complexity and mass transfer on microbial community structure

**Figure 7:**
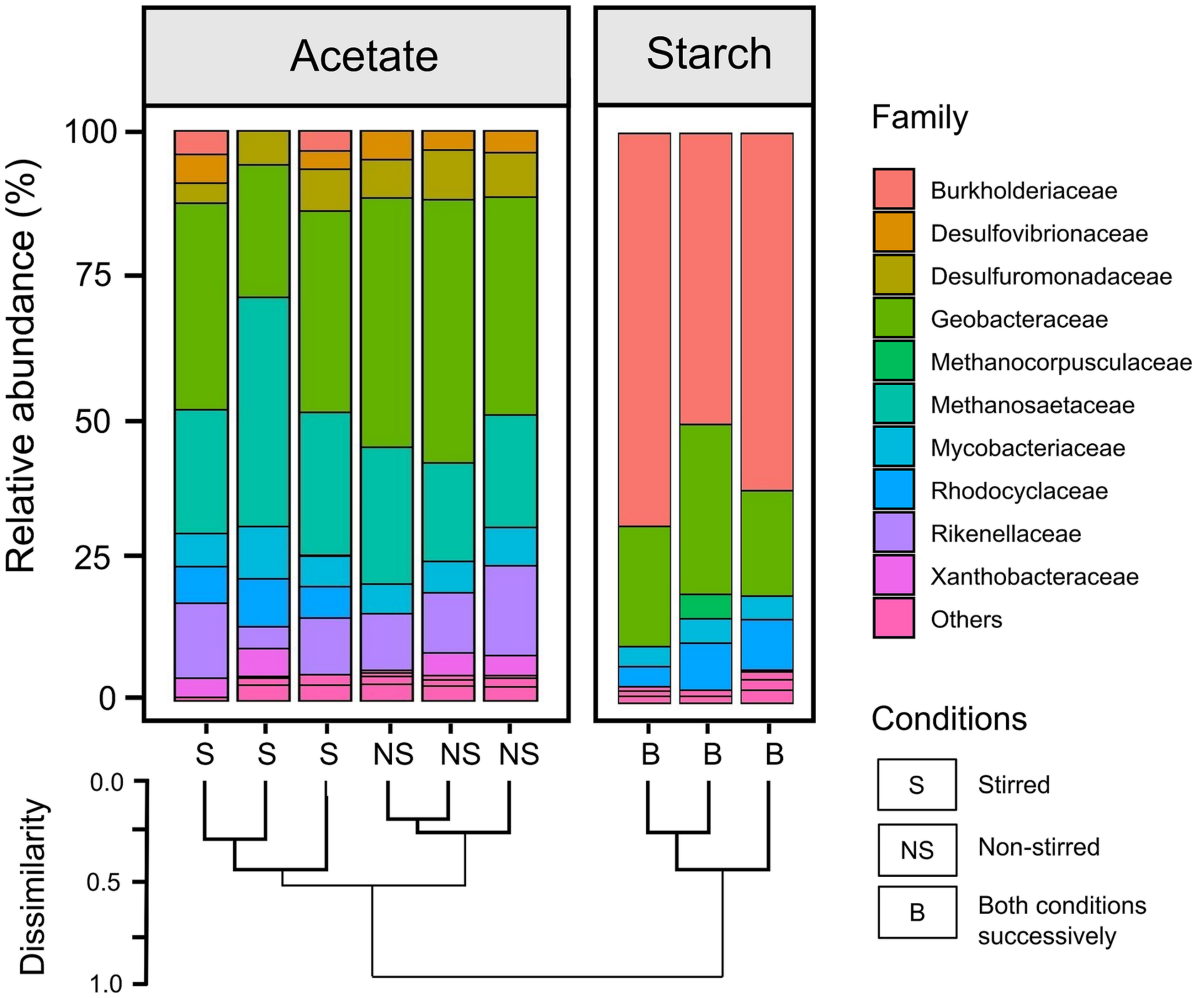
Bar chart showing the family-level relative abundance of the 16S rRNA community profile within the anodic biofilm of both acetate-fed and starch-fed MFCs. Starch-fed MFCs were run in three reactors successively, starting with stirred conditions followed by non-stirred conditions. A dendrogram illustrates hierarchical clustering of family abundance based on Bray-Curtis metrics. Due to the sequential stirring conditions for the starch-fed MFCs, only three biofilm samples were available for analysis, preventing a direct comparison between stirring conditions.

Microbial community analysis based on 16S rRNA gene sequencing was performed on the anodic biofilm samples for both acetate and starch-fed MFCs (post experiment). Notably, the relative abundance plots of the top 15 taxa (family-level) for each replicate demonstrated a high degree of similarity across all experimental conditions (Figure 7).

For acetate-fed MFCs, the relative abundance plots revealed a predominance of *Geobacteraceae* (12.3 ± 4.1% and 22.1 ± 1.8% in stirred and non-stirred MFCs, respectively) and *Methanosaeteae* (10.7 ± 2.5% in stirred and 9.9 ± 1.7% in non-stirred MFCs) in the anodic biofilm communities, as well as lower abundances of several sulfate reducing families (*Desulfovibrionaceae* and *Desulfuromonadaceae*) (Figure 7). As well as the differences in the relative abundances of *Geobacteraceae* in the anodic communities of the acetate MFCs, the family *Rhodocyclaceae* was present in the stirred MFCs but not in the non-stirred. The dendrogram in Figure 7 highlights distinct hierarchical clustering between the stirred and non-stirred acetate-fed communities. However, alpha diversity analysis indicates no significant difference in community diversity between the two conditions, with Shannon Diversity Index values of 4.8 ± 0.1 for stirred MFCs and 4.6 ± 0.1 for non-stirred MFCs (*p >* 0.05, *t*-test). Detailed alpha diversity plots are provided in Supplementary Figure S4.

The starch-fed anodic communities were dominated by both *Burkholderiaceae* (39.6 ± 7.4%) and *Geobacteraceae* (15.7 ± 6.8%) and appeared to have smaller abundances of other EAB such as the sulfate reducing families compared to the acetate anodic biofilms. The dendrogram shows clear differences between the microbial communities of the starch and acetate-fed MFCs (Figure 7). This observation is further supported by alpha diversity measurements which indicate that the starch-fed anodic biofilms were significantly less diverse, with a Shannon Diversity Index of 3.1 ± 0.1, compared to the acetate-fed anodic biofilms (*p* < 0.001, *t-*test for all three tested diversity indices; see Supplementary Figure S4).

Table 3 summarizes the key findings and differences in performance parameters observed across the four MFC conditions tested in this study. This comparison provides a clear overview of how each experimental condition influenced the system’s efficacy and offers insights into optimizing MFC design for complex wastewater treatment applications.

**Table 3:** Summary of key performance differences across the four MFC conditions.

Parameter		Acetate		Starch	
		Stirred	Non-stirred	Stirred	Non-stirred
Average time to acclimatize	(h)	~65	~65	~35	*
Average peak current density	(A/m^2^)	0.23 ± 0.02	0.29 ± 0.01	0.12 ± 0.02	0.15 ± 0.02
COD removal	(%)	73 ± 1.8	71 ± 3.3	47 ± 4.8	69 ± 1.2
Coulombic efficiency	(%)	38 ± 9.2	66 ± 9.1	19 ± 6.8	19 ± 3.9
Acetate consumption rate constant	(h)	0.0411 ± 0.0038	0.023 ± 0.0095	**	**
Hydrolysis-fermentation rate constant	(h)	N/A	N/A	0.0034 ± 0.0027	0.0045 ± 0.0013
Final fraction of electrons as current^a^	(%)	63.1 ± 9.4	105.3 ± 8.2	46.7 ± 8.9	62.8 ± 3.2
Final fraction of electrons as acetate^b^	(%)	5.8 ± 7.0	9.8 ± 8.3	7.6 ± 0.2	8.3 ± 1.2
Final fraction of electrons as “dead-end” VFAs^c^	(%)	6.3 ± 3.7	6.7 ± 3.4	44.4 ± 14.1	11.8 ± 4.9
Final fraction of unaccounted for electrons^d^	(%)	24.8 ± 2.8	0 ± 16.8	1.4 ± 5.3	17.1 ± 9.2
Fraction of COD consumed by aerobic acetate metabolism	(%)	15	1.8	9	1
*Geobacter* relative abundance	(%)	12.3 ± 4.1	22.1 ± 1.8	15.7 ± 6.8	
Alpha diversity (Shannon index)		4.8 ± 0.1	4.6 ± 0.1	3.1 ± 0.1	

Values represent the mean of replicates (acetate: *n* = 9, and starch: *n* = 6), with ± indicating the SD.

^*^Non-stirred starch-fed MFCs did not maintain stable currents during acclimatization.

^**^It was assumed that the rates of acetate consumption in the starch-fed MFCs were similar to those in acetate-fed MFCs.

^a–d Electron equivalent percentages totaling 100%.^

## 4 Discussion

This study is the first to explore the combined effects of substrate complexity and varying mass transfer conditions (stirring vs. non-stirring) on acclimatization rates, substrate degradation rates and the anodic microbial community selection in MFCs. The results show that substrate degradation in terms of rate and completeness was influenced by both complexity and mass transfer. Arguably, the most critical finding for the real-life application of this study was that under non-stirred conditions with a complex substrate, a successful electrogenic community failed to establish on the electrode. Previous studies have reported delays and reduced performance in glucose and starch-fed MFC’s compared to simpler substrates due to the challenges of forming complex communities required for the additional degradation steps (Lee et al., 2008; Ebadinezhad et al., 2019). However, our findings suggest there is greater complexity to this. The MFCs fed with a simple substrate (acetate) took approximately twice as long to acclimatize as those fed with starch, likely due to the initial scarcity and lag in the growth of acetate-consuming electrogens before exponential growth, as shown by current production. In the acetate-fed MFCs, stirring did not influence the acclimatization. In contrast, the MFCs fed with a complex substrate (starch) exhibited reduced lag times, indicating that the consortia of bacteria capable of metabolizing starch were more abundant. Stirring proved crucial here; by the end of the acclimatization period, all stirred MFCs acclimatized successfully, while none of the non-stirred MFCs produced a stable current. In practice, this suggests that large-scale reactors treating wastewater should be started under dynamic conditions to enhance performance.

It was hypothesized that stirring would enhance current production, substrate removal, and consequently coulombic efficiency (CE) by improving mass transfer of reactants within the system. This has been well demonstrated in literature (Yang et al., 2021; Lu et al., 2013). For example, in a tubular MFC, a rotating anode (an alternative mixing method) increased power outputs and COD removal when the speed of rotation increased (Pan et al., 2019). Interestingly, this was not observed in this present study. Both acetate and starch-fed MFCs under non-stirred conditions significantly outperformed the stirred systems. In the acetate-fed MFCs, the influence of mass transfer was evident when comparing the CE (a measure of the substrate’s efficiency in being converted into current). The non-stirred MFCs achieved a maximum CE of 66 ± 9.1%, significantly higher than the 38 ± 9.2% observed in stirred MFCs. Notably, these values surpass previously reported CEs for stirred acetate-fed MFCs using a comparable set-up (32%) (Velasquez-Orta et al., 2011). Counterintuitively, the stirred system where the biofilm had plentiful access to the food source, exhibited a significantly lower CE. Our findings show that 15% of the available COD in the stirred acetate-fed MFCs and 9% in the stirred starch-fed MFCs were lost to aerobic acetate metabolism, compared to only 1.8 and 1%, respectively, in the non-stirred systems. This was likely due to stirring increasing oxygen transfer at the membrane, leading to resource competition. This aligns with a previous study where MFC performance was significantly higher in a non-stirred system compared to a stirred system as a result of an increased redox potential in the anode (Min et al., 2005). The effect of oxygen intrusion on MFC performance has also been observed by Quan et al. (2012), where the presence of dissolved oxygen (0.1–4.0 mg/L) in the anode reduced the CE by 10%.

For starch-fed MFCs, the CE was consistently lower (19%) across both stirred and non-stirred conditions, aligning with previous reports (Velasquez-Orta et al., 2011). Stirring, however, lead to an increased diversion of electrons into “dead-end” VFAs, e.g., butyrate and propionate, which cannot be directly utilized by EAB like *Geobacter* (Miceli III et al., 2014; Tejedor-Sanz et al., 2018). These VFAs in the stirred system likely resulted from partial substrate breakdown due to competitive oxidation reactions, limiting current production. Additionally, stirring will have increased the contact between anodic bacteria and these “dead-end” VFAs, compounding their accumulation and reducing overall substrate removal. Findings from Choi et al. (2011) showed that the coexistence of VFAs can slow their individual removal, which may further explain the observed poor performance. These results suggest that stirring, or flow-through conditions in reactors (if an air-cathode is involved) may disadvantage current production, CE and in particular substrate removal, especially when using complex substrates.

Additional insights into the four MFC systems were obtained by mapping the electron balances. In the acetate-fed MFCs, periods of unaccounted-for electrons were observed, followed by their recovery. This indicates a potential electron storage in the system which was not accounted for in either the VFA measurement or the current, i.e., the biofilm itself acted as a capacitor, storing electrons in its matrix. This phenomenon was particularly evident in non-stirred acetate MFCs, where the electron balance exceeded 100%, suggesting storage built up during the initial acclimatization period. This supports earlier reports identifying biomass and residual organics as major electron sinks, after current, in glucose and acetate-fed systems (Lee et al., 2008), which our study shows could later serve as food sources to bacteria.

Kinetic analyzes revealed the first order hydrolysis-fermentation rates (*k* = 0.0034 ± 0.0027 h^−1^ for stirred; 0.0045 ± 0.0013 h^−1^ for non-stirred) were an order of magnitude lower than acetate consumption (combined electrogenesis and aerobic acetate metabolism) (*k* = 0.0411 ± 0.0038 h^−1^ for stirred; 0.023 ± 0.0095 h^−1^ for non-stirred). These are consistent with those previously reported by Velasquez-Orta et al. (2011) for a similar MFC set-up. The exact rate of the fermentation stage could not be calculated in this study as glucose could not be reliably measured. However, the fermentation rate calculated by Velasquez-Orta et al. (2011) (0.018 h^−1^) was an order of magnitude higher than the combined hydrolysis-fermentation rate (0.0024 h^−1^). We can therefore assume based on the findings of our study that the hydrolysis of complex substrates remains the rate-limiting step in these systems. Statistical analysis showed no significant differences (*p* > 0.05, *t*-test) in hydrolysis-fermentation rates between stirred and non-stirred conditions but revealed significant differences (*p* < 0.05, *t*-test) in acetate consumption rates, likely due to faster aerobic metabolisms. These findings suggest that aerobic metabolism can out compete electrogenic metabolism under acetate-fed conditions but not under starch-fed conditions.

Microbial community analyzes revealed a high degree of similarity in community composition across MFC replicates, with substrate type as the primary differentiating factor. This consistency is noteworthy, as microbial communities in experimental replicates typically diverge over time due to stochastic processes and ecological variability, even under controlled conditions (Estrela et al., 2021). For instance, studies by Leicester et al. (2023) and Zhou et al. (2013) reported substantial differences in microbial communities in both MFCs and MECs (microbial electrolysis cells) despite identical operational conditions. Acetate-fed MFCs demonstrated unexpectedly higher diversity than starch-fed MFCs, contrasting with the hypothesis that complex substrates foster broader communities (including hydrolytic, fermenting, and electrogenic bacteria) due to increased metabolic diversity requirements (Chae et al., 2009; Velasquez-Orta et al., 2011). Similar observations were reported by Heidrich et al. (2018) in acetate and wastewater-fed MFCs. These findings suggest that acetate, as an energy-rich and easily metabolisable substrate, may support the proliferation of acetate-feeding ‘weeds’, while complex substrates like starch may impose more challenging conditions, favoring smaller and more optimized communities. This counterintuitive result highlights the complexity of these systems, emphasizing the non-linear nature of microbial ecology in MFCs. The dominance of *Geobacteraceae* in the acetate-fed anodes aligns with previous reports of their role as key electrogens (Yates et al., 2012; Kan et al., 2011; Chae et al., 2009). However, the presence of methanogenic *Methanosaetaceae* highlights a competing metabolic pathway, where acetate is diverted to methanogenesis, a secondary reaction with faster kinetics than electrogenesis, reducing the MFC’s CE (Kaur et al., 2014). In the starch-fed MFCs, the predominance of *Burkholderiaceae* alongside *Geobacteraceae* suggests a potential synergistic role, with *Burkholderiaceae* mitigating oxygen intrusion by positioning themselves to face the bulk of the anolyte where bioavailability of oxygen can be achieved (Santoro et al., 2021). These findings reveal how community structure influences MFC performance, as oxygen-tolerant or facultative bacteria may alter substrate availability for EAB and could explain the reduced current generation in the starch-fed MFCs.

Stirring impacted microbial community composition without significantly affecting diversity in acetate-fed MFCs. Non-stirred systems exhibited a higher relative abundance of *Geobacteraceae*, suggesting that stable, low-shear environments favor electrogenic bacteria. Conversely, stirred systems displayed increased *Rhodocyclaceae* populations. This family, also observed in the starch-fed systems, includes facultative anaerobes known for their high tolerance to oxygen (Santoro et al., 2021). *Rhodocyclaceae* has been reported to utilize short-chain fatty acids and frequently dominates MFC anodes with open-air cathodes (Timmers et al., 2012). Notably, this family was absent in the non-stirred acetate-fed MFCs, reaffirming that stirring increased oxygen intrusion, which likely suppressed *Geobacteraceae* populations as both families competed for the same food source. These findings contrast with studies indicating that shear-stress-enriched biofilms enhance electrogenic populations and MFC performance (Yang et al., 2019). This discrepancy may result from differences in stirring intensity or oxygen exposure across studies.

Overall, this study highlights the intricate interplay between substrate complexity, mass transfer, and microbial community dynamics in MFCs. The findings reveal that substrate degradation was influenced by both substrate complexity and mass transfer conditions, with stirring proving essential for acclimatization in starch-fed MFCs. Stirring likely facilitated the development of microbial consortia capable of breaking down complex substrates. In contrast, acetate-fed MFCs acclimatized successfully under both stirred and non-stirred conditions, with the non-stirred MFCs achieving twice the relative abundance of electroactive *Geobacteraceae* and a higher CE (66% vs. 38%). Interestingly, stirring appeared to enhance oxygen intrusion into the anode, resulting in competition between facultative aerobes (e.g., *Rhodocyclaceae*) and electrogenic bacteria for resources. These findings highlight the trade-off between enhanced substrate availability and oxygen-induced competition. Microbial community analyzes provided further insights, revealing notable consistency among replicates, with substrate type being the primary driver of community structure. Unexpectedly, acetate-fed MFCs exhibited greater microbial diversity than starch-fed systems, which developed smaller and more specialized communities, counter to the expectations based on previous studies. Kinetic analyzes identified that hydrolysis of complex substrates remains the rate-limiting step in these systems, occurring at rates an order of magnitude slower than those of acetate consumption. While stirring had no significant effect on combined hydrolysis-fermentation rates, it significantly impacted acetate consumption rates, likely due to oxygen-driven competition.

For practical applications, the findings suggest that reactors treating wastewater with complex substrates (e.g., real wastewater) should begin under dynamic conditions to enhance acclimatization. During operation, however, maintaining non-stirred or minimally stirred conditions, especially when using air-cathodes, could improve coulombic efficiency and current production by reducing oxygen intrusion and electron diversion. Integrating MFCs with conventional wastewater treatment processes, such as anaerobic digestion (AD), presents a promising strategy to optimize substrate degradation and energy recovery, through overcoming the limitations of hydrolysis. This integration has shown efficiency in managing and reducing substrates with high organic loads, increasing energy conversion efficiencies, improving system longevity, and lowering operational costs, making it a promising approach for energy generation from wastewater treatment.

Despite these valuable insights, several limitations must be considered. This study was conducted under controlled laboratory conditions using only two substrates (acetate and starch) in batch-mode operation, which does not fully replicate the complexity of real wastewater environments. In real systems, the influx of diverse microbial communities and a wider range of substrates could introduce additional interactions that affect MFC performance. Additionally, the batch-mode setup does not reflect continuous-flow conditions, which are common in full-scale treatment systems and may impact microbial dynamics. Future research should focus on validating these findings in pilot-scale or continuous-flow systems, where environmental conditions and microbial populations are more dynamic. Investigating reactor designs that balance stirring and oxygen management, particularly at larger scales, and expanding the range of substrates tested to include more complex mixtures, representative of real-world wastewater, will further enhance the applicability of MFCs in practical wastewater treatment and energy recovery applications.

## Data Availability

The datasets presented in this study can be found in online repositories. The names of the repository/repositories and accession number(s) can be found below: https://www.ebi.ac.uk/ena, PRJEB80810.

## References

[ref1] Agrahari R. Bayar B. Abubackar H. N. Giri B. S. Rene E. R. Rani R. (2022). Advances in the development of electrode materials for improving the reactor kinetics in microbial fuel cells. Chemosphere 290:133184. doi: 10.1016/j.chemosphere.2021.133184 34890618

[ref2] Angelaalincy M. J. Navanietha Krishnaraj R. Shakambari G. Ashokkumar B. Kathiresan S. Varalakshmi P. (2018). Biofilm engineering approaches for improving the performance of microbial fuel cells and bioelectrochemical systems. Front. Energy Res. 6:63. doi: 10.3389/fenrg.2018.00063

[ref3] Apprill A. Mcnally S. Parsons R. Weber L. (2015). Minor revision to V4 region SSU rRNA 806R gene primer greatly increases detection of SAR11 bacterioplankton. Aquat. Microb. Ecol. 75, 129–137. doi: 10.3354/ame01753

[ref4] Ardakani M. N. Badalians Gholikandi G. (2020). Microbial fuel cells (MFCs) in integration with anaerobic treatment processes (AnTPs) and membrane bioreactors (MBRs) for simultaneous efficient wastewater/sludge treatment and energy recovery -a state-of-the-art review. Biomass Bioenergy 141:105726. doi: 10.1016/j.biombioe.2020.105726, PMID: 39678304

[ref5] Bajracharya S. (2020). “Microbial fuel cell coupled with anaerobic treatment processes for wastewater treatment” in Integrated Microbial Fuel Cells for Wastewater Treatment, 295–311.

[ref6] Bird H. Heidrich E. S. Leicester D. D. Theodosiou P. (2022). Pilot-scale microbial fuel cells (MFCs): a meta-analysis study to inform full-scale design principles for optimum wastewater treatment. J. Clean. Prod. 346:131227. doi: 10.1016/j.jclepro.2022.131227, PMID: 39678304

[ref7] Callahan B. J. McMurdie P. J. Rosen M. J. Han A. W. Johnson A. J. A. Holmes S. P. (2016). DADA2: high-resolution sample inference from Illumina amplicon data. Nat. Methods 13, 581–583. doi: 10.1038/nmeth.3869, PMID: 27214047 PMC4927377

[ref8] Cao Y. Mu H. Liu W. Zhang R. Guo J. Xian M.. (2019). Electricigens in the anode of microbial fuel cells: pure cultures versus mixed communities. Microbial Cell Factories 18, 1–14. doi: 10.1186/s12934-019-1087-z, PMID: 30782155 PMC6380051

[ref9] Caporaso J. G. Lauber C. L. Walters W. A. Berg-Lyons D. Lozupone C. A. Turnbaugh P. J.. (2011). Global patterns of 16S rRNA diversity at a depth of millions of sequences per sample. Proc. Natl. Acad. Sci. USA 108, 4516–4522. doi: 10.1073/pnas.1000080107, PMID: 20534432 PMC3063599

[ref10] Catal T. Li K. Bermek H. Liu H. (2008). Electricity production from twelve monosaccharides using microbial fuel cells. J. Power Sources 175, 196–200. doi: 10.1016/j.jpowsour.2007.09.083

[ref11] Chae K. J. Choi M. J. Lee J. W. Kim K. Y. Kim I. S. (2009). Effect of different substrates on the performance, bacterial diversity, and bacterial viability in microbial fuel cells. Bioresour. Technol. 100, 3518–3525. doi: 10.1016/j.biortech.2009.02.065, PMID: 19345574

[ref12] Choi J. D. R. Chang H. N. Han J. I. (2011). Performance of microbial fuel cell with volatile fatty acids from food wastes. Biotechnol. Lett. 33, 705–714. doi: 10.1007/s10529-010-0507-2, PMID: 21184134

[ref13] Christ O. Wilderer P. A. Angerhöfer R. Faulstich M. (2000). Mathematical modeling of the hydrolysis of anaerobic processes. Water Sci. Technol. 41, 61–65. doi: 10.2166/wst.2000.0056, PMID: 11382009

[ref14] Ebadinezhad B. Ebrahimi S. Shokrkar H. (2019). Evaluation of microbial fuel cell performance utilizing sequential batch feeding of different substrates. J. Electroanal. Chem. 836, 149–157. doi: 10.1016/j.jelechem.2019.02.004

[ref15] Energy Information Administration (2022) Residential energy consumption survey (RECS) [Online]. Available from: https://www.eia.gov/consumption/residential/ (Accessed November 29, 2024).

[ref16] Estrela S. Sánchez Á. Rebolleda-Gómez M. (2021). Multi-replicated enrichment communities as a model system in microbial ecology. Front. Microbiol. 12:657467. doi: 10.3389/fmicb.2021.657467 33897672 PMC8062719

[ref17] Godain A. Vogel T. M. Fongarland P. Haddour N. (2023). Influence of hydrodynamic forces on electroactive bacterial adhesion in microbial fuel cell anodes. Bioengineering 10:1380. doi: 10.3390/bioengineering10121380, PMID: 38135971 PMC10740411

[ref18] Godain A. Vogel T. M. Fongarland P. Haddour N. (2024). Influence of shear stress on electroactive biofilm characteristics and performance in microbial fuel cells. Biosens. Bioelectron. 244:115806. doi: 10.1016/j.bios.2023.115806 37944355

[ref19] Gude V. G. (2016). Wastewater treatment in microbial fuel cells – an overview. J. Clean. Prod. 122, 287–307. doi: 10.1016/j.jclepro.2016.02.022

[ref20] Heidrich E. S. Dolfing J. Wade M. J. Sloan W. T. Quince C. Curtis T. P. (2018). Temperature, inocula and substrate: contrasting electroactive consortia, diversity and performance in microbial fuel cells. Bioelectrochemistry 119, 43–50. doi: 10.1016/j.bioelechem.2017.07.006, PMID: 28910698

[ref21] Janicek A. Fan Y. Liu H. (2014). Design of microbial fuel cells for practical application: a review and analysis of scale-up studies. Biofuels 5, 79–92. doi: 10.4155/bfs.13.69

[ref22] Johnson E. Petersen T. Goeres D. M. (2021). Characterizing the shearing stresses within the CDC biofilm reactor using computational fluid dynamics. Microorganisms 9:1709. doi: 10.3390/microorganisms9081709, PMID: 34442788 PMC8399442

[ref23] Jones A. A. D. Buie C. R. (2019). Continuous shear stress alters metabolism, mass-transport, and growth in electroactive biofilms independent of surface substrate transport. Sci. Rep. 9, 1–8. doi: 10.1038/s41598-019-39267-2, PMID: 30796283 PMC6385357

[ref24] Jung S. Regan J. M. (2011). Influence of external resistance on Electrogenesis, Methanogenesis, and anode prokaryotic communities in microbial fuel cells. Appl. Environ. Microbiol. 77, 564–571. doi: 10.1128/AEM.01392-10, PMID: 21075886 PMC3020560

[ref25] Kan J. Hsu L. Cheung A. C. M. Pirbazari M. Nealson K. H. (2011). Current production by bacterial communities in microbial fuel cells enriched from wastewater sludge with different electron donors. Environ. Sci. Technol. 45, 1139–1146. doi: 10.1021/es102645v, PMID: 21171663

[ref26] Kannaiah Goud R. Venkata Mohan S. (2011). Pre-fermentation of waste as a strategy to enhance the performance of single chambered microbial fuel cell (MFC). Int. J. Hydrog. Energy 36, 13753–13762. doi: 10.1016/j.ijhydene.2011.07.128

[ref27] Kaur A. Boghani H. C. Michie I. Dinsdale R. M. Guwy A. J. Premier G. C. (2014). Inhibition of methane production in microbial fuel cells: operating strategies which select electrogens over methanogens. Bioresour. Technol. 173, 75–81. doi: 10.1016/j.biortech.2014.09.091, PMID: 25285762

[ref29] Lee H. S. Parameswaran P. Kato-Marcus A. Torres C. I. Rittmann B. E. (2008). Evaluation of energy-conversion efficiencies in microbial fuel cells (MFCs) utilizing fermentable and non-fermentable substrates. Water Res. 42, 1501–1510. doi: 10.1016/j.watres.2007.10.036, PMID: 18035391

[ref30] Leicester D. (2020). *Energy recovery from a high strength waste stream using pilot-scale bioelectrochemical systems* [Online]. Newcastle upon Tyne: Newcastle University.

[ref31] Leicester D. D. Settle S. McCann C. M. Heidrich E. S. (2023). Investigating variability in microbial fuel cells. Appl. Environ. Microbiol. 89:e0218122. doi: 10.1128/aem.02181-22, PMID: 36840599 PMC10057029

[ref32] Logan B. (2008). Microbial Fuel Cells. Hoboken, New Jersey: John Wiley & Sons.

[ref33] Logan B. E. (2009). Exoelectrogenic bacteria that power microbial fuel cells. Nat. Rev. Microbiol. 7, 375–381. doi: 10.1038/nrmicro2113 19330018

[ref34] Lu S. S. Zhao Y. G. Liu R. (2013). Effect of different operating conditions on MFC performance. Adv. Mater. Res. 724-725, 762–768. doi: 10.4028/www.scientific.net/AMR.724-725.762

[ref35] Mata-Alvarez J. Macé S. Llabrés P. (2000). Anaerobic digestion of organic solid wastes. An overview of research achievements and perspectives. Bioresour. Technol. 74, 3–16. doi: 10.1016/S0960-8524(00)00023-7

[ref36] McMurdie P. J. Holmes S. (2013). Phyloseq: an R package for reproducible interactive analysis and graphics of microbiome census data. PLoS One 8:e61217. doi: 10.1371/journal.pone.0061217, PMID: 23630581 PMC3632530

[ref37] Metcalf W. Eddy C. (2003). Wastewater engineering: Treatment and reuse. New York: McGraw-Hill.

[ref38] Miceli J. F. III Garcia-Peña I. Parameswaran P. Torres I. Krajmalnik-Brown R. (2014). Combining microbial cultures for efficient production of electricity from butyrate in a microbial electrochemical cell. Bioresour. Technol. 169, 169–174. doi: 10.1016/j.biortech.2014.06.090 25048958 PMC4284095

[ref39] Milligan A. (2023) Decision decision on revised typical domestic consumption values for gas and electricity and economy 7 consumption split.

[ref40] Min B. Kim J. R. Oh S. E. Regan J. M. Logan B. E. (2005). Electricity generation from swine wastewater using microbial fuel cells. Water Res. 39, 4961–4968. doi: 10.1016/j.watres.2005.09.039, PMID: 16293279

[ref41] Pan Y. Zhu T. He Z. (2019). Energy advantage of anode electrode rotation over anolyte recirculation for operating a tubular microbial fuel cell. Electrochem. Commun. 106:106529. doi: 10.1016/j.elecom.2019.106529

[ref42] Pandey P. Shinde V. N. Deopurkar R. L. Kale S. P. Patil S. A. Pant D. (2016). Recent advances in the use of different substrates in microbial fuel cells toward wastewater treatment and simultaneous energy recovery. Appl. Energy 168, 706–723. doi: 10.1016/j.apenergy.2016.01.056

[ref43] Pant D. Van Bogaert G. Diels L. Vanbroekhoven K. (2010). A review of the substrates used in microbial fuel cells (MFCs) for sustainable energy production. Bioresour. Technol. 101, 1533–1543. doi: 10.1016/j.biortech.2009.10.017, PMID: 19892549

[ref44] Parada A. E. Needham D. M. Fuhrman J. A. (2016). Every base matters: assessing small subunit rRNA primers for marine microbiomes with mock communities, time series and global field samples. Environ. Microbiol. 18, 1403–1414. doi: 10.1111/1462-2920.13023, PMID: 26271760

[ref45] Patil S. A. Hägerhäll C. Gorton L. Patil S. A. Hägerhäll C. Gorton L. (2012). Electron transfer mechanisms between microorganisms and electrodes in bioelectrochemical systems. Bioanal. Rev. 1, 159–192. doi: 10.1007/11663_2013_2

[ref46] Prathiba S. Kumar P. S. Vo D. V. N. (2022). Recent advancements in microbial fuel cells: a review on its electron transfer mechanisms, microbial community, types of substrates and design for bio-electrochemical treatment. Chemosphere 286:131856. doi: 10.1016/j.chemosphere.2021.131856 34399268

[ref47] Quan X. Quan Y. P. Tao K. (2012). Effect of anode aeration on the performance and microbial community of an air-cathode microbial fuel cell. Chem. Eng. J. 210, 150–156. doi: 10.1016/j.cej.2012.09.009

[ref48] Santoro C. Babanova S. Cristiani P. Artyushkova K. Atanassov P. Bergel A.. (2021). How comparable are microbial electrochemical systems around the globe? An electrochemical and microbiological cross-laboratory study. ChemSusChem 14, 2313–2330. doi: 10.1002/cssc.202100294, PMID: 33755321 PMC8252665

[ref49] Sonawane J. M. Mahadevan R. Pandey A. Greener J. (2022). Recent progress in microbial fuel cells using substrates from diverse sources. Heliyon 8:e12353. doi: 10.1016/j.heliyon.2022.e12353, PMID: 36582703 PMC9792797

[ref50] Sonawane J. M. Yadav A. Ghosh P. C. Adeloju S. B. (2017). Recent advances in the development and utilization of modern anode materials for high performance microbial fuel cells. Biosens. Bioelectron. 90, 558–576. doi: 10.1016/j.bios.2016.10.014, PMID: 27825877

[ref51] Tchobanoglous G. Burton F. L. Stensel D. H. (2003). Wastewater engineering: Treatment and reuse Boston: Metcalf & Edy Inc - McGraw Hill.

[ref52] Tejedor-Sanz S. Fernández-Labrador P. Hart S. Torres C. I. Esteve-Núñez A. (2018). Geobacter dominates the inner layers of a stratified biofilm on a fluidized anode during brewery wastewater treatment. Front. Microbiol. 9:378. doi: 10.3389/fmicb.2018.00378 29568284 PMC5853052

[ref53] Thygesen A. Poulsen F. W. Min B. Angelidaki I. Thomsen A. B. (2009). The effect of different substrates and humic acid on power generation in microbial fuel cell operation. Bioresour. Technol. 100, 1186–1191. doi: 10.1016/j.biortech.2008.07.067, PMID: 18815026

[ref54] Timmers R. A. Strik D. P. B. T. B. Arampatzoglou C. Buisman C. J. N. Hamelers H. V. M. (2012). Rhizosphere anode model explains high oxygen levels during operation of a *Glyceria maxima* PMFC. Bioresour. Technol. 108, 60–67. doi: 10.1016/j.biortech.2011.10.088, PMID: 22265596

[ref55] Torres C. I. Kato Marcus A. Rittmann B. E. (2007). Kinetics of consumption of fermentation products by anode-respiring bacteria. Appl. Microbiol. Biotechnol. 77, 689–697. doi: 10.1007/s00253-007-1198-z, PMID: 17909786

[ref56] Trapero J. R. Horcajada L. Linares J. J. Lobato J. (2017). Is microbial fuel cell technology ready? An economic answer towards industrial commercialization. Appl. Energy 185, 698–707. doi: 10.1016/j.apenergy.2016.10.109

[ref57] Vavilin V. A. Rytov S. V. Lokshina L. Y. Rintala J. A. Lyberatos G. (2001). Simplified hydrolysis models for the optimal design of two-stage anaerobic digestion. Water Res. 35, 4247–4251. doi: 10.1016/S0043-1354(01)00148-8, PMID: 11791857

[ref58] Velasquez-Orta S. B. Yu E. Katuri K. P. Head I. M. Curtis T. P. Scott K. (2011). Evaluation of hydrolysis and fermentation rates in microbial fuel cells. Appl. Microbiol. Biotechnol. 90, 789–798. doi: 10.1007/s00253-011-3126-5, PMID: 21347728

[ref59] Yang J. Cheng S. Li C. Sun Y. Huang H. (2019). Shear stress affects biofilm structure and consequently current generation of bioanode in microbial electrochemical systems (MESS). Front. Microbiol. 10:398. doi: 10.3389/fmicb.2019.00398, PMID: 30894842 PMC6415583

[ref60] Yang W. Li J. Fu Q. Zhang L. Wei Z. Liao Q.. (2021). Minimizing mass transfer losses in microbial fuel cells: theories, progresses and prospectives. Renew. Sust. Energ. Rev. 136:110460. doi: 10.1016/j.rser.2020.110460

[ref61] Yates M. D. Kiely P. D. Call D. F. Rismani-Yazdi H. Bibby K. Peccia J.. (2012). Convergent development of anodic bacterial communities in microbial fuel cells. ISME J. 6, 2002–2013. doi: 10.1038/ismej.2012.42, PMID: 22572637 PMC3475369

[ref62] Yilmaz P. Parfrey L. W. Yarza P. Gerken J. Pruesse E. Quast C.. (2014). The SILVA and “all-species living tree project (LTP)” taxonomic frameworks. Nucleic Acids Res. 42, D643–D648. doi: 10.1093/nar/gkt1209, PMID: 24293649 PMC3965112

[ref63] Zafar H. Ishaq S. Peleato N. Roberts D. (2022). Meta-analysis of operational performance and response metrics of microbial fuel cells (MFCs) fed with complex food waste. J. Environ. Manag. 315:115152. doi: 10.1016/j.jenvman.2022.115152 35525044

[ref64] Zhang L. Gao R. Naka A. Hendrickx T. L. G. Rijnaarts H. H. M. Zeeman G. (2016). Hydrolysis rate constants at 10–25 °C can be more than doubled by a short anaerobic pre-hydrolysis at 35 °C. Water Res. 104, 283–291. doi: 10.1016/j.watres.2016.07.038 27551780

[ref65] Zhang F. Ge Z. Grimaud J. Hurst J. He Z. (2013). Long-term performance of liter-scale microbial fuel cells treating primary effluent installed in a municipal wastewater treatment facility. Environ. Sci. Technol. 47, 4941–4948. doi: 10.1021/es400631r, PMID: 23517192

[ref66] Zhang Y. Min B. Huang L. Angelidaki I. (2011). Electricity generation and microbial community response to substrate changes in microbial fuel cell. Bioresour. Technol. 102, 1166–1173. doi: 10.1016/j.biortech.2010.09.044, PMID: 20952193

[ref67] Zhao F. Heidrich E. S. Curtis T. P. Dolfing J. (2020). The effect of anode potential on current production from complex substrates in bioelectrochemical systems: a case study with glucose. Appl. Microbiol. Biotechnol. 104, 5133–5143. doi: 10.1007/s00253-020-10547-6, PMID: 32248443 PMC7228986

[ref68] Zhong D. Zhu K. Ma W. Li J. Li K. Dai C. (2021). Optimization of a completely mixed anaerobic biofilm reactor (CMABR), based on brewery wastewater treatment. Water 13:606. doi: 10.3390/w13050606

[ref69] Zhou J. Liu W. Deng Y. Jiang Y. H. Xue K. He Z.. (2013). Stochastic assembly leads to alternative communities with distinct functions in a bioreactor microbial community. MBio 4:e00584-12. doi: 10.1128/mBio.00584-12, PMID: 23462114 PMC3585448

[ref70] Zou S. He Z. (2018). Efficiently “pumping out” value-added resources from wastewater by bioelectrochemical systems: a review from energy perspectives. Water Res. 131, 62–73. doi: 10.1016/j.watres.2017.12.026, PMID: 29274548

