# Supplementary material for: Understanding the limitations of substrate degradation in bioelectrochemical systems
Source: Front Microbiol. 2025 Jan 6;15:1511142. doi: 10.3389/fmicb.2024.1511142 (PMC11743565; doi:10.3389/fmicb.2024.1511142)
Supplement: Supplementary file 1 [file Data_Sheet_1.PDF]

Supplementary Material

S.1 Electron equivalents of organic substrates

Table 1: Electron equivalents of organic substrates and degradation intermediates and end products (Zhao et al., 2020b) (Rittmann & McCarty, 2020).

Molecules	Degradation reaction	e^- eq per carbon
Glucose	$C_6H_{12}O_6 + 6H_2O \rightarrow 6CO_2 + 24H^+ + 24e^-$	24/6 = 4
Propionate	$C_3H_6O_2 + 4H_2O \rightarrow 3CO_2 + 14H^+ + 14e^-$	14/3 = 4.67
Ethanol	$C_2H_6O + 3H_2O \rightarrow 2CO_2 + 12H^+ + 12e^-$	12/2 = 6
Acetate	$C_2H_4O_2 + 2H_2O \rightarrow 2CO_2 + 8H^+ + 8e^-$	8/2 = 4
Formate	$CH_2O_2 \rightarrow CO_2 + 2H^+ + 2e^-$	2/1 = 2
Butyrate	$C_4H_8O_2 + 6H_2O \rightarrow 4CO_2 + 20H^+ + 20e^-$	20/4 = 4
Valerate	$C_5H_9O_2 + 8H_2O \rightarrow 5CO_2 + 25H^+ + 25e^-$	25/5 = 5

Table 2: Distribution of electron equivalents (%) for the four different MFC conditions at each sample point.

	Time of sample (h)	Average electron balance (%)									
		Acetic acid	Formic acid	Propionic acid	Butyric acid	Isobutyric acid	Valeric acid	Isovaleric acid	Current	Total	Unknown
Acetate - stirred	**0**	100.0 ± 0.0	0.0 ± 0.0	0.0 ± 0.0	0.0 ± 0.0	0.0 ± 0.0	0.0 ± 0.0	0.0 ± 0.0	0.0 ± 0.0	100.0 ± 0.0	0.0 ± 0.0
	**1**	96.3 ± 2.5	0.6 ± 0.5	1.4 ± 2.5	0.0 ± 0.0	0.0 ± 0.0	0.0 ± 0.0	0.0 ± 0.0	0.2 ± 0.2	98.5 ± 5.2	1.5 ± 5.2
	**2.5**	95.6 ± 3.0	0.5 ± 0.6	4.1 ± 1.2	0.0 ± 0.0	0.0 ± 0.0	0.0 ± 0.0	0.0 ± 0.0	1.1 ± 0.3	101.3 ± 4.2	0 ± 4.2
	**3.5**	92.0 ± 5.2	0.5 ± 0.6	1.6 ± 2.1	0.0 ± 0.0	0.0 ± 0.0	0.0 ± 0.0	0.0 ± 0.0	1.8 ± 0.2	95.8 ± 6.6	4.2 ± 6.6
	**4.5**	88.7 ± 2.9	0.5 ± 0.6	1.7 ± 2.2	0.0 ± 0.0	0.0 ± 0.0	0.0 ± 0.0	0.0 ± 0.0	2.6 ± 0.1	93.4 ± 4.9	6.6 ± 4.9
	**24**	39.4 ± 4.8	0.4 ± 0.6	3.1 ± 0.9	0.7 ± 0.7	0.2 ± 0.0	0.0 ± 0.0	0.0 ± 0.0	15.8 ± 1.3	59.6 ± 6.1	40.4 ± 6.1
	**26**	32.2 ± 2.6	0.4 ± 0.7	2.1 ± 0.7	0.0 ± 0.0	0.2 ± 0.0	0.0 ± 0.0	0.0 ± 0.0	17.2 ± 1.4	52.3 ± 3.4	47.7 ± 3.4
	**28**	25.8 ± 6.5	0.6 ± 0.6	1.9 ± 1.5	0.0 ± 0.0	0.2 ± 0.0	0.0 ± 0.0	0.0 ± 0.0	18.7 ± 1.7	47.2 ± 7.1	52.8 ± 7.1
	**48**	4.4 ± 0.4	0.2 ± 0.4	0.9 ± 0.7	0.0 ± 0.0	1.7 ± 0.1	0.0 ± 0.0	0.0 ± 0.0	29.6 ± 2.8	36.8 ± 3.0	63.2 ± 3.0
	**51**	2.7 ± 1.3	0.4 ± 0.7	0.4 ± 0.7	0.0 ± 0.0	1.7 ± 0.1	0.0 ± 0.0	0.0 ± 0.0	31.7 ± 3.0	36.9 ± 3.8	63.1 ± 3.8
	**71**	4.6 ± 2.3	0.4 ± 0.6	1.1 ± 1.1	0.0 ± 0.0	1.7 ± 0.0	0.0 ± 0.0	0.0 ± 0.0	37.8 ± 5.6	45.6 ± 3.2	54.4 ± 3.2
	**96**	5.3 ± 2.4	0.4 ± 0.6	1.4 ± 1.1	0.0 ± 0.0	1.0 ± 0.0	0.0 ± 0.0	0.0 ± 0.0	45.7 ± 8.1	53.6 ± 4.1	46.4 ± 4.1
	**160**	5.8 ± 7.0	0.3 ± 0.6	2.2 ± 2.9	0.0 ± 0.0	3.8 ± 0.1	0.0 ± 0.0	0.0 ± 0.0	63.1 ± 9.4	75.2 ± 2.8	24.8 ± 2.8
Acetate – non-stirred	**0**	100.0 ± 0.0	0.0 ± 0.0	0.0 ± 0.0	0.0 ± 0.0	0.0 ± 0.0	0.0 ± 0.0	0.0 ± 0.0	0.0 ± 0.0	100.0 ± 0.0	0.0 ± 0.0
	**1**	98.2 ± 2.9	0.5 ± 0.5	1.4 ± 2.4	0.0 ± 0.0	0.0 ± 0.0	0.0 ± 0.0	0.0 ± 0.0	0.1 ± 0.2	100.3 ± 4.9	0.0 ± 4.9
	**2.5**	97.6 ± 1.4	0.5 ± 0.6	3.3 ± 1.8	0.0 ± 0.0	0.0 ± 0.0	0.0 ± 0.0	0.0 ± 0.0	1.0 ± 0.5	102.3 ± 3.3	0.0 ± 3.3
	**3.5**	96.1 ± 2.6	0.4 ± 0.6	2.1 ± 2.0	0.0 ± 0.0	0.0 ± 0.0	0.0 ± 0.0	0.0 ± 0.0	1.7 ± 0.5	100.3 ± 4.8	0.0 ± 4.8
	**4.5**	95.9 ± 2.0	0.4 ± 0.7	2.6 ± 2.2	0.0 ± 0.0	0.0 ± 0.0	0.0 ± 0.0	0.0 ± 0.0	2.7 ± 0.4	101.5 ± 4.5	0.0 ± 4.5
	**24**	63.1 ± 12.7	1.2 ± 1.2	4.1 ± 1.6	0.0 ± 0.0	0.2 ± 0.0	0.0 ± 0.0	0.0 ± 0.0	19.8 ± 0.8	88.3 ± 14.9	11.7 ±14.9
	**26**	55.1 ± 14.2	1.0 ± 1.0	3.8 ± 2.3	0.4 ± 0.7	0.2 ± 0.0	0.0 ± 0.0	0.0 ± 0.0	21.5 ± 1.0	82.0 ± 16.6	18.0 ±16.6
	**28**	50.6 ± 9.1	0.5 ± 0.6	3.2 ± 2.1	0.1 ± 0.2	0.2 ± 0.0	0.0 ± 0.0	0.0 ± 0.0	23.3 ± 1.2	77.9 ± 10.7	22.1 ±10.7
	**48**	26.9 ± 9.1	1.1 ± 1.0	2.4 ± 1.4	0.0 ± 0.0	1.4 ± 0.1	0.0 ± 0.0	0.0 ± 0.0	38.8 ± 2.9	70.5 ± 11.0	29.5 ±11.0
	**51**	25.6 ± 10.1	2.4 ± 3.0	5.2 ± 7.3	0.0 ± 0.0	1.6 ± 0.1	0.0 ± 0.0	0.0 ± 0.0	42.0 ± 3.2	76.7 ± 17.2	23.3±17.2
	**71**	13.3 ± 4.7	1.1 ± 1.1	1.8 ± 0.9	0.0 ± 0.0	1.6 ± 0.2	0.0 ± 0.0	0.0 ± 0.0	57.6 ± 3.4	75.4 ± 6.7	24.6±6.7
	**96**	10.9 ± 5.5	0.9 ± 0.8	1.2 ± 1.1	0.0 ± 0.0	1.1 ± 0.2	0.0 ± 0.0	0.0 ± 0.0	73.0 ± 3.4	87.1 ± 6.8	12.9± 6.8
	**160**	9.8 ± 8.3	0.7 ± 0.6	2.3 ± 3.1	0.0 ± 0.0	3.7 ± 0.6	0.0 ± 0.0	0.0 ± 0.0	105.3 ± 8.2	121.8 ± 16.8	0.0± 16.8

**Starch- stirred**	**0**	2.7 ± 3.8	0.6 ± 0.8	1.3 ± 1.9	0.0 ± 0.0	1.6 ± 1.6	0.0 ± 0.0	0.0 ± 0.0	0.0 ± 0.0	100.0 ± 0.0	0.0 ± 0.0
	**1**	12.2 ± 4.7	0.2 ± 0.3	7.1 ± 5.5	1.2 ± 1.6	8.4 ± 2.9	0.5 ± 0.6	0.5 ± 0.6	0.3 ± 0.1	**30.2** ± 15.6	69.8 ±15.6
	**2.5**	13.4 ± 3.4	0.3 ± 0.5	6.5 ± 5.1	1.1 ± 1.5	8.7 ± 2.4	0.7 ± 0.9	0.7 ± 0.9	0.6 ± 0.2	**32.1** ± 13.8	67.9 ±13.8
	**3.5**	15.5 ± 8.6	0.1 ± 0.1	8.3 ± 7.6	1.0 ± 1.4	9.2 ± 1.0	0.6 ± 0.9	0.6 ± 0.9	1.3 ± 0.1	**36.7** ± 21.0	63.3 ±21.0
	**4.5**	14.4 ± 8.0	0.0 ± 0.0	7.2 ± 7.3	1.1 ± 1.6	11.4 ± 0.2	0.7 ± 0.9	0.7 ± 0.9	1.8 ± 0.1	**37.1** ± 18.5	62.9 ±18.5
	**24**	31.3 ± 18.2	0.9 ± 1.0	11.2 ±11.9	2.6 ± 3.7	17.4 ± 1.6	0.5 ± 0.7	0.5 ± 0.7	9.1 ± 0.9	**73.4** ± 36.9	26.6 ±36.9
	**26**	31.7 ± 18.6	0.8 ± 1.0	11.9 ±11.8	2.2 ± 3.2	15.9 ± 0.3	0.3 ± 0.5	0.3 ± 0.5	9.8 ± 0.9	**73.1** ± 34.3	26.9 ±34.3
	**28**	32.1 ± 19.1	0.8 ± 1.2	12.6 ±12.6	2.1 ± 3.0	15.7 ± 0.2	0.3 ± 0.4	0.3 ± 0.4	10.4 ± 1.0	**74.4** ± 35.6	25.6 ±35.6
	**48**	32.7 ± 10.1	0.8 ± 1.0	20.2 ±12.2	2.5 ± 3.0	18.2 ± 2.2	0.3 ± 0.4	0.3 ± 0.4	15.7 ± 2.3	**90.8** ± 22.6	9.2 ± 22.6
	**51**	27.7 ± 6.4	1.1 ± 0.9	23.0 ±12.4	3.4 ± 2.1	18.6 ± 2.6	0.4 ± 0.5	0.4 ± 0.5	18.8 ± 3.1	**93.4** ± 17.0	6.6 ± 17.0
	**71**	22.8 ± 2.7	1.3 ± 0.8	25.8 ±12.7	4.3 ± 1.1	19.0 ± 3.1	0.5 ± 0.5	0.5 ± 0.5	22.0 ± 3.9	**96.0** ± 11.2	4.0 ± 11.2
	**96**	10.7 ± 2.3	0.4 ± 0.5	22.4 ± 5.2	3.5 ± 0.7	25.0 ± 4.2	0.7 ± 0.6	0.7 ± 0.6	28.3 ± 5.0	**91.6** ± 2.1	8.4 ± 2.1
	**160**	7.6 ± 0.2	0.3 ± 0.5	21.0 ±12.2	2.6 ± 0.6	18.7 ± 1.5	0.9 ± 0.7	0.9 ± 0.7	46.7 ± 8.9	**98.6** ± 5.3	1.4 ± 5.3
**Starch – non-stirred**	**0**	11.1 ± 6.1	2.9 ± 4.1	5.9 ± 8.3	0.0 ± 0.0	4.1 ± 5.8	0.0 ± 0.0	0.0 ± 0.0	0.0 ± 0.0	**100.0** ± 0.0	0.0 ±0.0
	**1**	11.0 ± 4.9	1.5 ± 2.2	3.1 ± 3.9	0.0 ± 0.0	5.2 ± 2.5	0.0 ± 0.0	0.0 ± 0.0	0.3 ± 0.3	**21.2** ± 13.2	78.8 ±13.2
	**2.5**	13.2 ± 3.6	1.7 ± 2.3	4.7 ± 4.8	0.0 ± 0.0	10.9 ± 2.9	0.0 ± 0.0	0.0 ± 0.0	0.8 ± 0.4	**31.3** ± 13.3	68.7 ±13.3
	**3.5**	14.7 ± 5.4	1.9 ± 2.7	4.8 ± 5.0	0.0 ± 0.0	10.3 ± 0.3	0.0 ± 0.0	0.0 ± 0.0	1.7 ± 0.4	**33.4** ± 12.4	66.6 ±12.4
	**4.5**	11.5 ± 0.6	0.3 ± 0.2	2.7 ± 1.5	0.0 ± 0.0	7.9 ± 2.6	0.0 ± 0.0	0.0 ± 0.0	2.3 ± 0.5	**24.6** ± 2.0	75.4 ± 2.0
	**24**	34.4 ± 4.7	1.2 ± 1.1	10.8 ± 2.7	0.0 ± 0.0	13.0 ± 7.5	0.0 ± 0.0	0.0 ± 0.0	11.8 ± 0.8	**71.0** ± 0.2	29.0 ± 0.2
	**26**	36.7 ± 7.0	0.2 ± 0.2	12.8 ± 4.6	0.0 ± 0.0	13.5 ± 8.9	0.0 ± 0.0	0.0 ± 0.0	12.7 ± 0.9	**75.9** ± 1.9	24.1 ± 1.9
	**28**	36.1 ± 5.5	0.3 ± 0.3	13.0 ± 3.8	0.0 ± 0.0	14.6 ± 10.7	0.0 ± 0.0	0.0 ± 0.0	13.6 ± 0.9	**77.5** ± 2.0	22.5 ± 2.0
	**48**	19.3 ± 1.7	0.7 ± 0.2	12.1 ± 0.7	0.4 ± 0.5	12.5 ± 6.4	0.0 ± 0.0	0.0 ± 0.0	22.0 ± 0.9	**66.9** ± 9.0	33.1 ± 9.0
	**51**	14.6 ± 2.4	0.5 ± 0.3	10.6 ± 0.7	0.1 ± 0.2	12.0 ± 6.6	0.0 ± 0.0	0.0 ± 0.0	26.7 ± 1.2	**64.5** ± 11.4	35.5 ±11.4
	**71**	9.9 ± 3.1	0.4 ± 0.5	9.1 ± 2.0	0.3 ± 0.4	11.5 ± 6.8	0.0 ± 0.0	0.0 ± 0.0	31.4 ± 1.5	**62.6** ± 14.4	37.4 ±14.4
	**96**	9.4 ± 0.7	0.8 ± 0.4	4.2 ± 2.8	0.0 ± 0.0	9.3 ± 7.0	0.0 ± 0.0	0.0 ± 0.0	41.3 ± 2.5	**65.0** ± 12.2	35.0 ±12.2
	**160**	8.3 ± 1.2	0.7 ± 0.3	2.1 ± 2.0	0.0 ± 0.0	9.0 ± 2.6	0.0 ± 0.0	0.0 ± 0.0	62.8 ± 3.2	**82.9** ± 9.2	17.1 ± 9.2

S.2 Rate calculations for the degradation pathways

Rates were calculated by constructing a linear plot of the time (first 28 h) vs the natural logarithm (ln) of the reactant concentration (Figure 1) based on Equation 1.

The linear plots below were used to obtain the kinetic rate constant *k:*

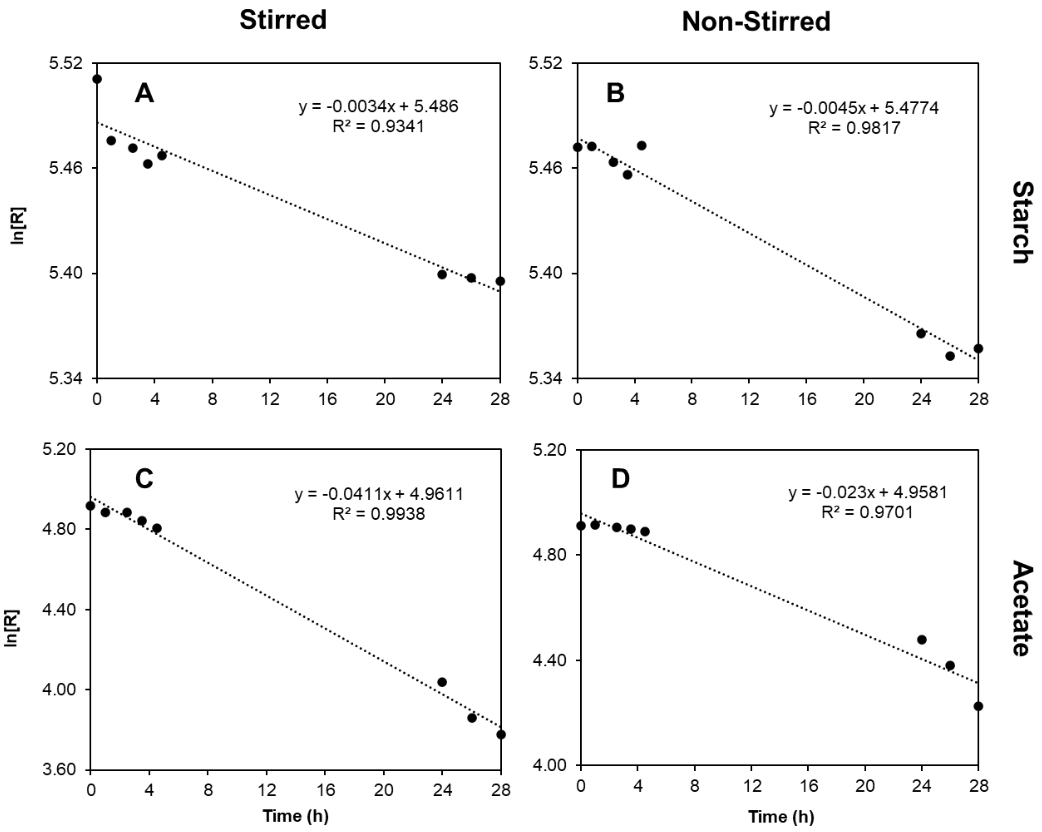

Figure 1: *Linear plots of time vs the natural logarithm of the reactant concentration for:* ***(A)*** *starch-fed MFCs under stirred conditions;* ***(B)*** *starch-fed MFCs under non-stirred conditions;* ***(C)*** *acetate-fed MFCs under stirred conditions; and* ***(D)*** *acetate-fed MFCs under non-stirred conditions.*

The hydrolysis-fermentation pathway (conversion of starch to acetate) was calculated as follows:

- Stirred conditions

$$\ln(\text{starch}) = -0.0034t + 5.486 \qquad [1]$$

$$k = 0.0034\ h^{-1}$$

- Non-stirred conditions

$$\ln(\text{starch}) = -0.0045t + 5.4774 \qquad [1]$$

$$k = 0.0045\ h^{-1}$$

Rates of acetate consumption/electrogenesis was calculated as follows:

- Stirred conditions

$$\ln(\text{acetate}) = -0.0411t + 4.9611 \qquad [1]$$

$$k = 0.0411\ h^{-1}$$

- Non-stirred conditions

$$\ln(\text{acetate}) = -0.0232t + 4.9581 \qquad [1]$$

$$k = 0.0232\ h^{-1}$$

S.3 Total VFA production/consumption for individual batch runs

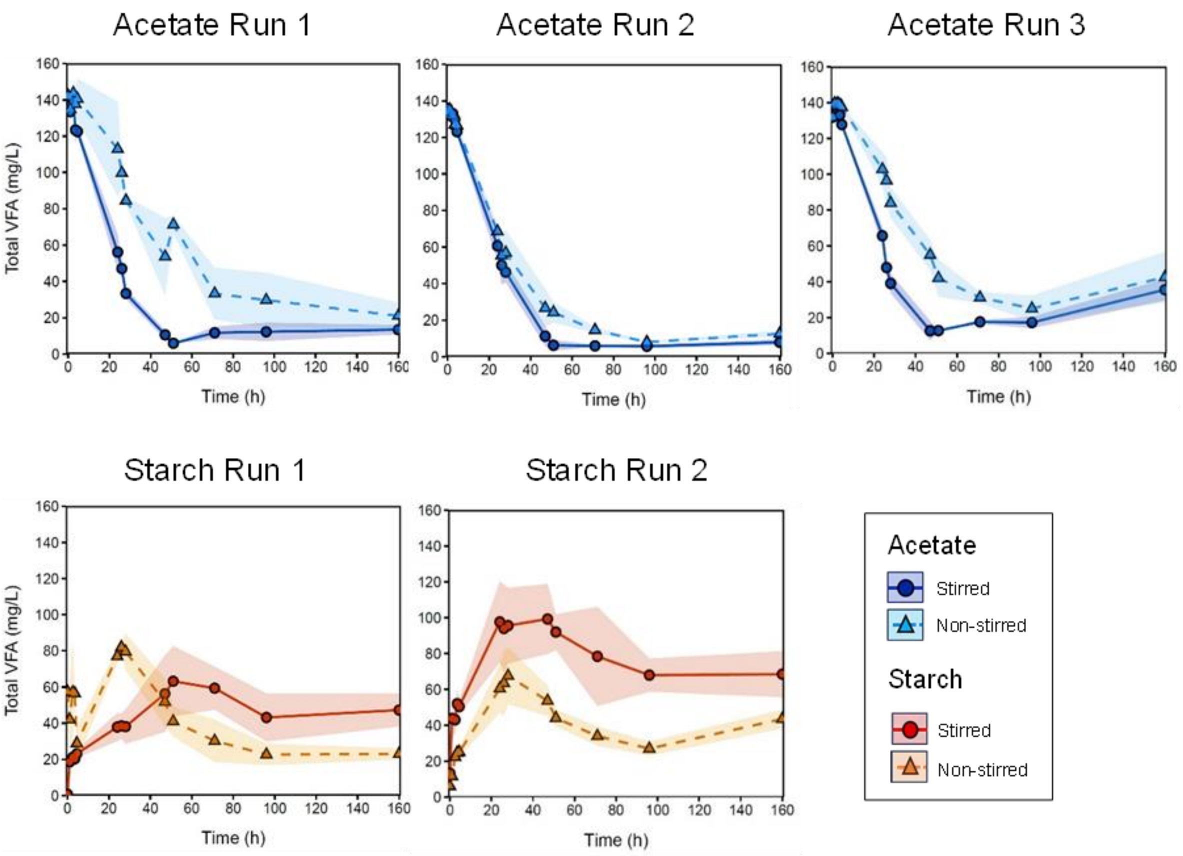

Figure 2: *Total VFA production/consumption during all batch runs for acetate and starch-fed MFCs.* Values represent the average of replicates (n=3). The error bands denote SD.

S.4 Oxygen intrusion measurements

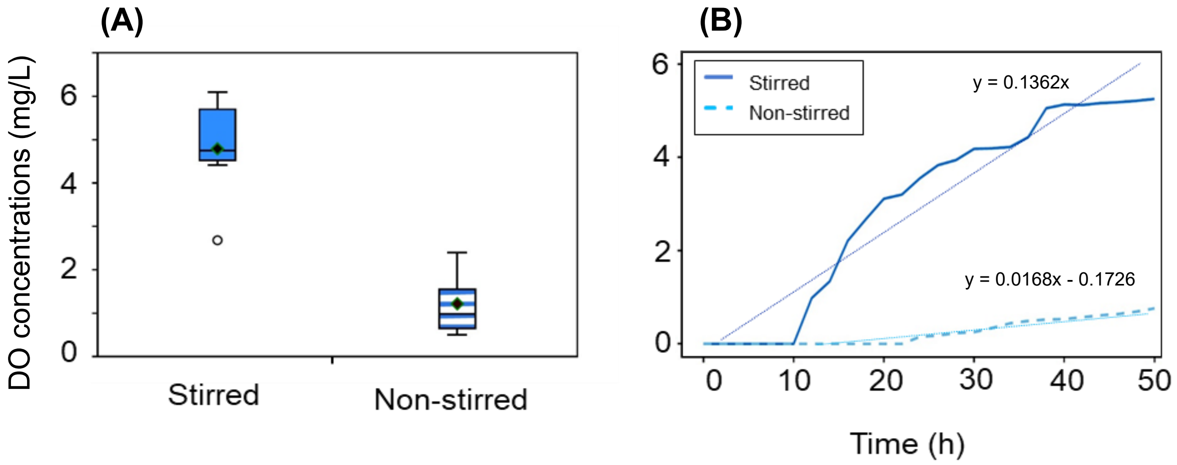

Figure 3: (A) *Effluent dissolved oxygen (DO) concentrations for both acetate and starch-fed MFCs under stirred and non-stirred conditions.* ***(B)*** *Continuous DO concentrations of a stirred and non-stirred MFC with phosphate buffer.*

The amount of COD consumed by incoming oxygen was calculated based on the following calculation taken from Heidrich et al., (2011):

$$COD + O_2 \rightarrow CO_2 + H_2O$$

Therefore, 1g of COD is equal to 1/32 mol oxygen (1 mol of O_2 for every 32g of COD).

- Reactor volume 0.33 L
- Length on batch runs: 160 hours
- Acetate MFC influent: 150 mg/LCOD
- Starch MFC influent: 250 mg/LCOD

COD (in g) in MFCs:

$$Acetate = \frac{150 \times 0.33}{1000} = 0.0495\ g$$

$$Starch = \frac{250 \times 0.33}{1000} = 0.0825\ g$$

S.4.1 Stirred MFC calculations

Rate of oxygen intrusion = 0.14 mg/L of oxygen per hour (gradient of line taken from equation, Figure 3B).

Rate of O_2 entering the system

$$= \frac{1.4 \times 10^{-4} g/L/h \times 0.33\ L}{32\ g/mol} = 1.44 \times 10^{-6}\ mol/h$$

O_2 available in batch run

$$= 1.44 \times 10^{-6}\ mol/h\ \times 160\ h\ = 2.3 \times 10^{-4}\ moles$$

COD available for O_2 consumption

$$= 2.3 \times 10^{-4} mol \times 32 = 0.0074\ g\ COD$$

Stirred acetate:

Percentage of COD available for O_2 to consume

$$= \left(\frac{0.0074\ g}{0.0495\ g}\right) \times 100\ = \mathbf{15}\%$$

Stirred starch:

Percentage of COD available for O_2 to consume $= \left(\frac{0.00825\ g}{0.0495\ g}\right) \times 100 = \mathbf{9}\%$

S.4.2 Non-stirred MFC calculations

Rate of oxygen intrusion = 0.017 mg/L of oxygen per hour (gradient of line taken from equation, Figure 3B).

Rate of O_2 entering the system $= \frac{1.7 \times 10^{-5} g/L/h \times 0.33\ L}{32\ g/mol} = 1.75 \times 10^{-7}\ mol/h$

O_2 available in batch run $= 1.75 \times 10^{-7}\ mol/h\ \times 160\ h\ = 2.8 \times 10^{-5}\ moles$

COD available for O_2 consumption $= 2.8 \times 10^{-5} mol \times 32 = 0.0009\ g\ COD$

Non-stirred acetate:

Percentage of COD available for O_2 to consume $= \left(\frac{0.0009\ g}{0.0495\ g}\right) \times 100 = \mathbf{1.8}\%$

Non-stirred starch:

Percentage of COD available for O_2 to consume $= \left(\frac{0.0009\ g}{0.0495\ g}\right) \times 100 = \mathbf{1}\%$

S.5 Alpha diversity analysis

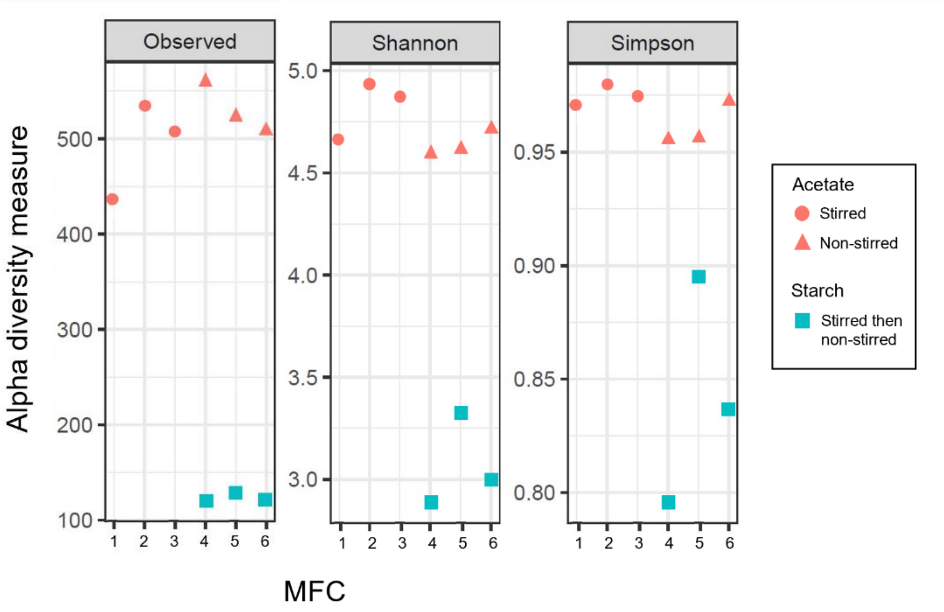

Figure 4: *Alpha diversity plots showing the diversity of the anodic microbial communities in the acetate and starch-fed MFCs, based on observed species, Shannon and Simpson indices. Starch-fed MFCs, which underwent sequential stirring conditions (stirred, then non-stirred), included only three biofilm samples for analysis (denoted by square icons).*
